# Non-destructive identification of closely related japonica rice seeds using hyperspectral imaging combined with HHO-optimized XGBoost

**DOI:** 10.3389/fpls.2026.1909987

**Published:** 2026-09-17

**Authors:** Chunxu Jiang, Shixue Gu, Gang Liu, Yang Liu, Rongjie Huang, Shaozhong Song

**Affiliations:** 1 School of Computer Science, Baicheng Normal University, Baicheng, China; 2 Haiming Primary School, Baicheng, China; 3 School of Data Science and Artificial Intelligence, Jilin Engineering Normal University, Changchun, China

**Keywords:** closely related japonica rice seeds, hyperspectral imaging, machine learning, non-destructive identification, smart agriculture

## Abstract

Genetically closely-related japonica rice seed varieties are difficult to distinguish using conventional identification techniques. These traditional methods generally require time-consuming operations, complex experimental workflows, and seed destruction for sample testing. Hyperspectral imaging offers a feasible non-destructive way to screen crop germplasm resources. Nevertheless, raw hyperspectral signals are easily disturbed by instrument noise and external environmental interference; the dataset also suffers high dimensionality and redundant spectral features, which hinders model performance. Standard XGBoost models rely on manual hyperparameter adjustment, which frequently falls into local optimal solutions. Besides, poor model interpretability makes it hard to reduce classification errors between genetically similar rice materials. To address these practical problems, this work constructed an improved XGBoost framework combined with multi-step spectral preprocessing and Harris Hawks Optimization (HHO). SHAP analysis was further introduced to reveal classification rules of critical spectral bands and realize accurate, non-destructive identification of rice seeds. Five pixel statistical calculation strategies were compared, and mean spectral curves were chosen as model input data. Savitzky-Golay smoothing and Min-Max normalization were implemented to suppress spectral noise. The Successive Projections Algorithm (SPA) was then adopted to pick out 22 sensitive bands with low multicollinearity. This step preserved effective discriminative information while lowering feature dimensions. SHAP values were applied to quantify feature contributions from both global importance and single-sample perspectives, alleviating the black-box limitation of machine-learning classifiers. Nine different swarm intelligence algorithms were tested for automatic hyperparameter searching of XGBoost. The final HHO-XGBoost approach achieved 94.72% overall accuracy and a Kappa coefficient of 0.9389. Per-class F1-score results demonstrated that the established method could obviously decrease misclassification among nine japonica rice varieties. This technical workflow integrates spectral preprocessing, feature screening, interpretive analysis and intelligent parameter optimization. It can serve as a practical reference for non-destructive identification of crop germplasm with close genetic relationships.

## 1 Introduction

Japonica rice is one of the vital food crops worldwide, and the varietal purity of its seeds directly governs field agronomic traits, grain yield and agricultural product quality (Meng et al., 2022). Closely related japonica rice germplasms usually share highly similar genetic backgrounds with trivial morphological distinctions, which greatly increases the risk of variety adulteration (Yang et al., 2023; Zhang et al., 2021; Xu et al., 2022). Therefore, rapid and precise identification of closely related japonica rice seeds acts as a critical prerequisite for seed quality control, germplasm resource conservation and the popularization of elite varieties. Traditional seed identification methods mainly consist of field phenotypic observation, biochemical detection and molecular biological assays. Field identification requires a long experimental cycle, and the final results are susceptible to environmental and artificial interferences. By contrast, physicochemical and molecular detection methods are destructive, accompanied by complicated operational procedures and high testing costs, which are not suitable for high-throughput rapid screening of bulk seed samples (Frischie et al., 2020). Accordingly, it is urgent to develop a non-destructive, efficient and convenient seed variety recognition technique to provide technical support for quality inspection in the seed industry.

In recent years, with the rapid advancement of artificial intelligence technology, deep learning methods have been widely applied to hyperspectral image classification due to their powerful automatic feature learning capability (Song et al., 2025; Yu et al., 2024; Bi et al., 2022; Li et al., 2024; Yu et al., 2025). Relying on multi-layer nonlinear architectures, deep learning models can hierarchically extract abstract deep features without manual feature engineering and achieve excellent classification performance with large-scale labeled datasets (Bao and Bambil, 2021). However, such strategies still exhibit inherent limitations in the non-destructive detection of crop germplasm, especially for the identification of closely related rice germplasms. First, deep learning models contain massive parameters and require substantial annotated samples for adequate training. In practical breeding scenarios, the collection and labeling of closely related japonica rice seed samples are labor-intensive and costly (Bayazit et al., 2025; Rahaman et al., 2025). Under limited sample conditions, deep learning models are prone to overfitting, resulting in degraded generalization performance and poor adaptability to small-scale germplasm identification tasks. Second, mainstream deep learning frameworks are typical black-box models (Jin et al., 2025). Although they can yield high classification accuracy, they cannot explicitly screen critical sensitive spectral bands or establish interpretable correlations between spectral responses and seed physicochemical properties, including moisture, protein, and starch contents. This deficiency restricts the mechanistic interpretation of confusing spectral characteristics among genetically similar varieties. Moreover, deep learning training heavily relies on high-performance GPU resources and involves time-consuming iterative optimization, making lightweight deployment difficult for conventional field detection devices and limiting its applicability in large-scale, rapid seed screening scenarios (Akbarpour et al., 2025; Chang et al., 2025). In addition, the complex hyperparameter systems of deep learning models lead to experience-dependent tuning procedures and low experimental reproducibility (Huang et al., 2022). In contrast, machine learning algorithms represented by XGBoost possess simpler structures, lower data and hardware requirements, and superior stability for limited-sample tasks. Combined with spectral preprocessing, sensitive band selection, and SHAP-based interpretability analysis, XGBoost can not only maintain reliable classification accuracy for closely related japonica rice seeds but also quantitatively reveal the contribution mechanism of spectral features. Further integrated with swarm intelligence optimization for global hyperparameter searching, this framework is more suitable for the precise identification of highly similar crop germplasm in practical seed detection scenarios.

Hyperspectral imaging technology can simultaneously acquire spatial image information and continuous spectral data of samples within the visible and near-infrared bands (Fu et al., 2025; Tejasree and Agilandeeswari, 2024; Zhao et al., 2025). It is capable of capturing subtle spectral variations derived from internal physicochemical components of seeds, including moisture, protein, and starch, thereby enabling non-destructive, rapid, and multi-index synchronous detection (Zhao et al., 2026; Hu et al., 2025; Zhang et al., 2023). Benefiting from these superiorities, hyperspectral imaging has been widely adopted in crop seed quality evaluation, variety classification, and pest and disease identification (Gui et al., 2025; Li et al., 2025; Gu et al., 2024; Zhang et al., 2024). In the hyperspectral identification of japonica rice seeds, raw hyperspectral data typically suffer from high dimensionality, excessive redundant information, and prominent noise interference induced by instrumental errors and illumination fluctuations (Ge et al., 2024; Diao et al., 2023). Direct modeling using original spectral data not only increases computational consumption but also easily causes overfitting and misclassification of closely related varieties, severely restricting the recognition accuracy and generalization performance of classification models (Bi et al., 2024). Although conventional spectral preprocessing and feature selection methods can optimize datasets to a certain extent, comparative investigations on the effectiveness of different denoising and feature screening strategies for the identification of closely related japonica rice germplasms remain insufficient (Diao et al., 2023). Furthermore, traditional machine learning models generally rely on manual grid search for hyperparameter tuning, which hardly obtains optimal parameter combinations and tends to fall into local optima, resulting in a performance ceiling in germplasm classification tasks.

XGBoost has exhibited promising application potential in crop hyperspectral classification tasks due to its outstanding fitting capability and robust anti-overfitting performance (Xiao et al., 2024; Sun et al., 2025; Yuan et al., 2024). Nevertheless, its core hyperparameters, including learning rate, maximum tree depth, and the number of base learners, substantially determine the final classification performance, and conventional manual tuning suffers from strong subjectivity and low optimization efficiency. As an advanced swarm intelligence algorithm, Harris Hawks Optimization (HHO) dynamically balances global exploration and local exploitation based on prey escape energy, effectively avoiding the premature convergence defect of traditional optimization approaches and enabling efficient searching for optimal hyperparameter combinations in high-dimensional parameter spaces (Shehab et al., 2022). The Successive Projections Algorithm (SPA) can screen sensitive spectral bands with minimal multicollinearity, compress feature dimensions while retaining critical discriminative information for variety identification (de Souza et al., 2025). Combined with SHAP interpretability analysis, it further reveals the internal mechanism between spectral features and classification results, addressing the inherent black-box limitation of machine learning models (Liang et al., 2025; Yu et al., 2025).

Considering the existing research limitations mentioned above, nine genetically similar japonica rice seed varieties were chosen as experimental materials in the present work. Such rice germplasms share high genetic homology and cannot be easily separated through ordinary phenotypic observation, which makes them a typical difficult sample set for germplasm preservation and seed identification tasks. Hyperspectral imaging was utilized to acquire spectral information from individual seed samples. We compared several pixel-based statistical approaches to identify the most suitable spectral representation. Different preprocessing routines were also tested to select an effective noise-removal solution, and normalization was subsequently applied to remove magnitude discrepancies across different spectral bands. After evaluating several dimension-reduction and band-selection techniques, SPA was used to obtain key sensitive spectral features. SHAP analysis was then carried out to clarify feature importance and uncover the underlying classification mechanism. Using XGBoost as the base classification model, we assessed the hyperparameter-searching capability of nine swarm intelligence algorithms. An HHO-tuned XGBoost model was ultimately built to distinguish these hard-to-separate japonica rice seeds. Step-by-step experiments were designed to quantify how much performance gain could be achieved via spectral denoising, normalization, feature selection and intelligent parameter optimization. This work develops a high-accuracy hyperspectral identification approach for genetically close japonica rice germplasm. It expects to offer new technical ideas and methodological support for large-scale non-destructive variety discrimination and fast purity testing of rice seeds.

## 2 Materials and methods

### 2.1 Data set preparation

#### 2.1.1 Rice samples

In this study, nine varieties of closely related japonica rice seeds with high genetic similarity were selected as experimental materials, which were provided by the Rice Research Institute of Jilin Academy of Agricultural Sciences. A total of 1800 seed samples were collected, with approximately 200 grains for each variety, to construct the hyperspectral dataset. As shown in **Figure 1**, the nine japonica rice varieties exhibit extremely similar morphological characteristics in grain shape, color, and size (Chen et al., 2025). The subtle phenotypic differences lead to low distinguishability, resulting in low efficiency and high error rates in manual identification. Meanwhile, such high visual similarity also imposes great difficulties on accurate classification using conventional computer vision methods. Accordingly, hyperspectral imaging is adopted to explore inherent spectral differences hidden in visually indistinguishable seeds, enabling efficient and non-destructive identification of closely related japonica rice germplasms.

**Figure 1:**
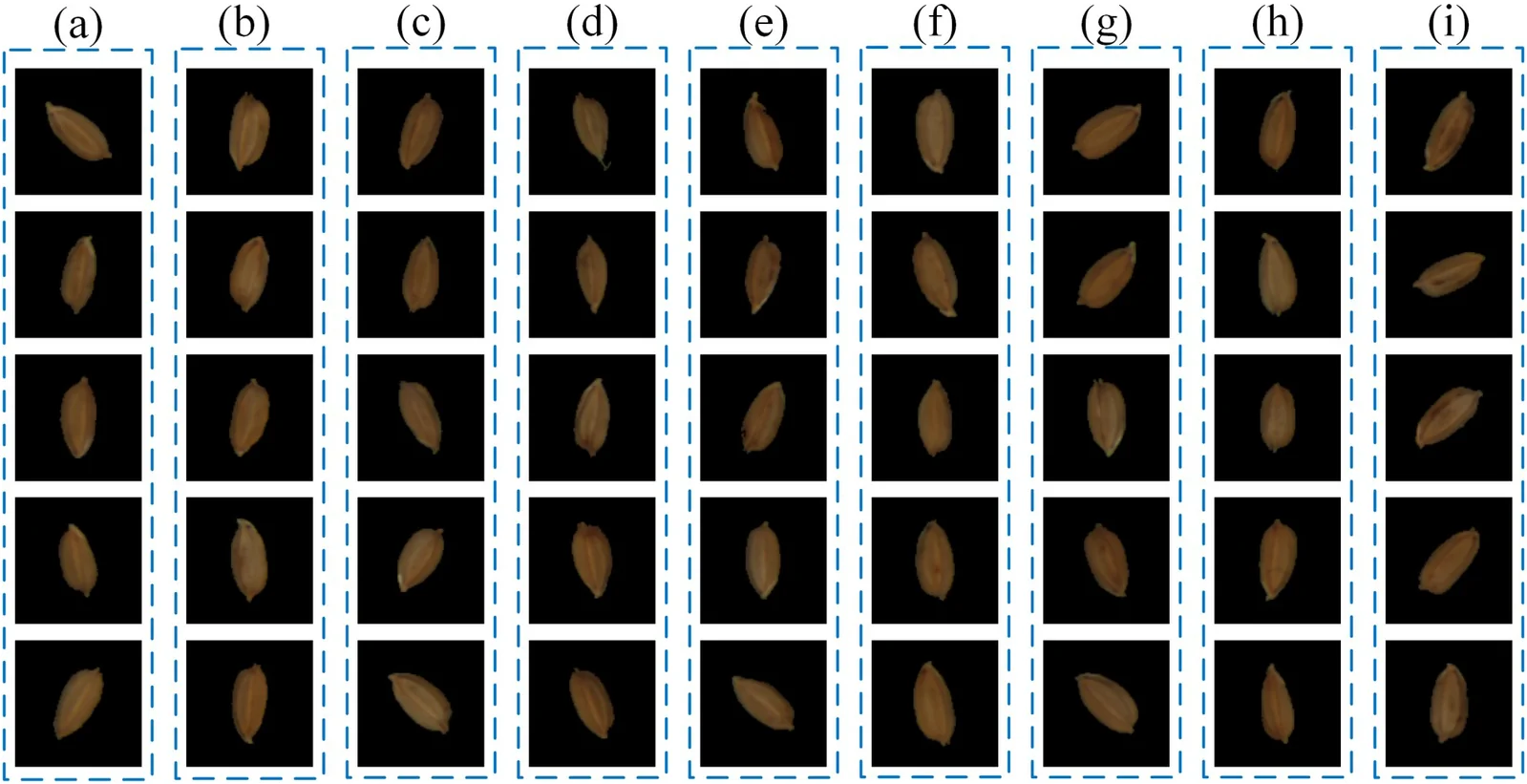
Visual presentation of different japonica rice seed varieties. **(a)** Jijing 809, **(b)** Jijing 812, **(c)** Jijing 816, **(d)** Jijing 821, **(e)** Jijing 825, **(f)** Jijing 826, **(g)** Jijing 827, **(h)** Jijing 830, **(i)** Jijing 851. Correspondingly, the labels from 1 to 9 were assigned to varieties **(a–i)** during model training.

#### 2.1.2 Hyperspectral image acquisition and calibration

The configuration of the hyperspectral imaging system used in this study is illustrated in **Figure 2**. The experimental setup mainly consists of a hyperspectral camera, a uniform illumination module, an electric linear translation stage, a power supply, and a computer terminal equipped with SpectrononPro acquisition software. The hyperspectral sensor employed is Specim FX10 (Specim, Oulu, Finland). The spectrometer covers an effective spectral range of 392.38–1011.01 nm with 462 continuous spectral bands. No spectral bands were removed at both ends of the spectrum; all acquired bands were retained for subsequent analysis after radiometric calibration. Radiometric normalization was implemented using a polytetrafluoroethylene (PTFE) white reference panel with a nominal reflectance of 99%. The slit width was set to 50 μm, providing a spatial resolution of 0.15 mm per pixel and a spectral resolution of 1.3 nm. Such high-resolution spectral acquisition capability enables the system to capture subtle spectral differences among closely related japonica rice seeds, providing high-quality original data for subsequent feature mining and classification modeling.

**Figure 2:**
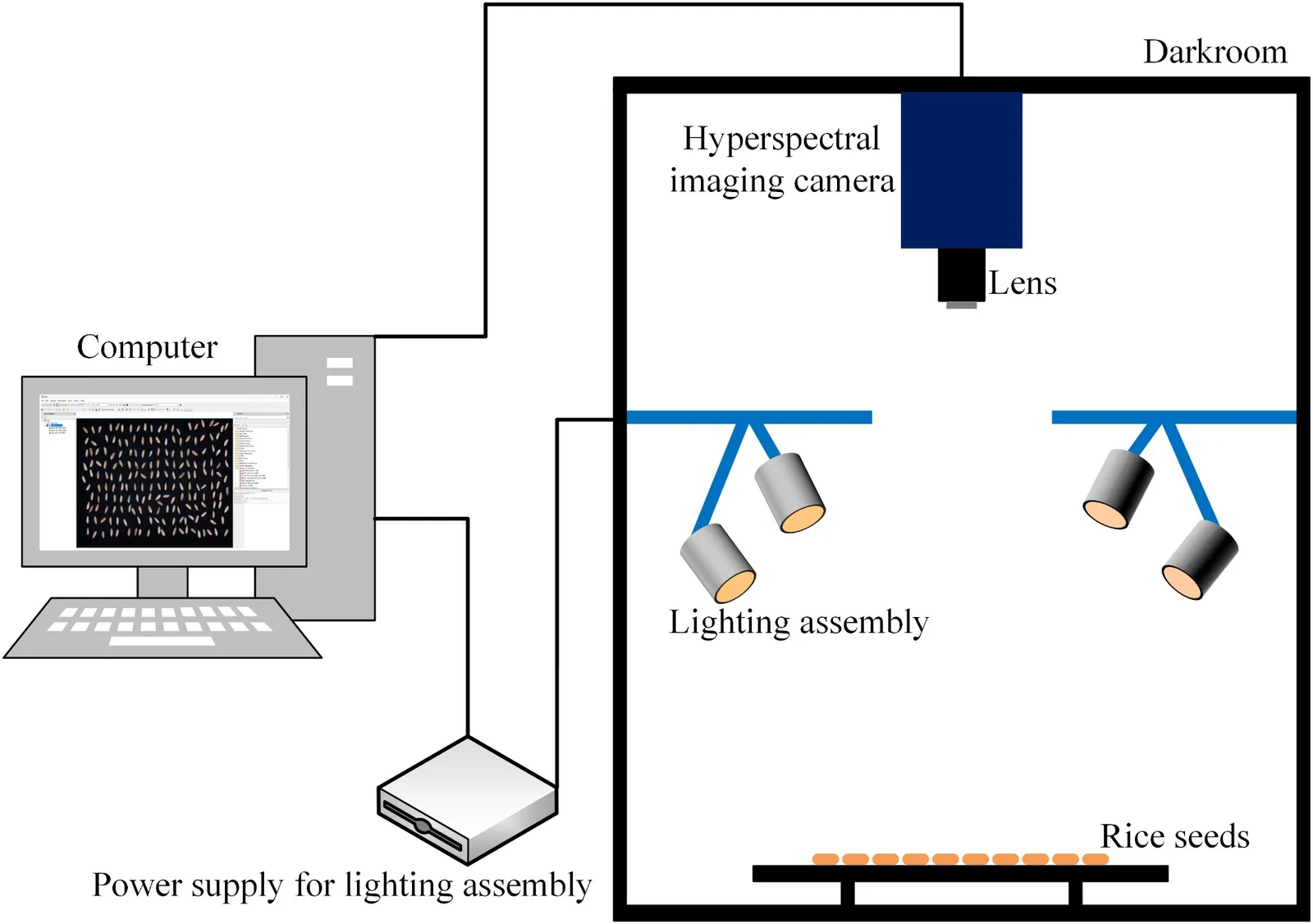
Schematic diagram of a hyperspectral imaging system.

During data acquisition, 200 seeds were randomly selected for each japonica rice variety and evenly placed in a customized 10 × 20 sampling template. All seeds were arranged without overlap or adhesion. The template was placed on a black electric translation stage, and hyperspectral images were acquired through uniform scanning. Following the identical acquisition procedure, hyperspectral data of all nine rice varieties were sequentially collected to construct the complete original dataset.

Due to the inherent dark current noise of the imaging sensor, the gray values of raw images cannot directly reflect the real spectral reflectance characteristics of seed samples. Therefore, black-and-white correction was performed to eliminate system noise and uneven illumination interference. The original digital grayscale values were converted into standardized spectral reflectance values within the range of 0–1, and the corresponding correction formula is shown in Equation 1.

(1) $$I_{c}=\frac{I_{o}-I_{b}}{I_{w}-I_{b}}$$

Where *I_c_* represents the corrected standard hyperspectral image after black-and-white calibration; *I_o_* denotes the original raw hyperspectral image of rice seeds acquired during the experiment; *I_b_* is the dark reference image collected with the lens fully covered, which is used to eliminate the dark current noise of the sensor; *I_w_* refers to the white reference image obtained by scanning a standard whiteboard, which is applied to normalize and correct the uneven illumination intensity.

#### 2.1.3 Hyperspectral feature extraction

Spectral information extraction techniques for hyperspectral images have been widely employed in the non-destructive detection of food and agricultural products. In hyperspectral data analysis, spectral features are generally extracted based on the manually delineated Region of Interest (ROI). The two most commonly used extraction strategies are pixel-level spectrum extraction and mean spectrum extraction, which exhibit distinct advantages and applicability for different analytical scenarios.

Pixel-level spectrum extraction acquires spectral information from all pixels within the ROI, retaining detailed spectral variations across the sample surface. This method is suitable for quantitative analysis and visual characterization of the spatial distribution of chemical components in heterogeneous samples and shows unique superiority in local feature detection. However, for crop seed classification tasks, rice seeds exhibit inherent structural and compositional heterogeneity, resulting in inconsistent spectral responses among different surface pixels of a single seed. Such pixel-level spectral discrepancies do not correspond to varietal differences and may introduce redundant feature interference, thereby increasing the risk of model misclassification and limiting its applicability for closely related seed identification.

In contrast, mean spectrum extraction treats each seed as an independent analytical unit by averaging all pixel spectra within the individual seed ROI. This approach fully accommodates the natural structural and compositional heterogeneity of seed grains and can stably and accurately represent the overall spectral characteristics of a single seed. Unlike traditional point-based near-infrared spectrometers, which only collect spectral information from limited surface points and fail to reflect comprehensive seed physicochemical properties, hyperspectral imaging combined with mean spectrum extraction can effectively capture holistic seed spectral features and overcome the limitations of conventional spectral detection.

Furthermore, pixel-level spectral data are highly susceptible to sensor noise, environmental interference, and seed surface morphological variations, including shape curvature and surface flatness, resulting in low signal-to-noise ratio and substantial random spectral fluctuations. In comparison, mean spectrum calculation suppresses random noise through multi-pixel averaging, smooths spectral profiles, and significantly improves data stability and effectiveness, which is beneficial for enhancing the final classification performance. Therefore, mean spectrum extraction has been widely adopted in agricultural spectral detection and classification research.

In this study, the detailed workflow of seed mean spectrum extraction was implemented as follows. First, image segmentation was performed on the calibrated hyperspectral images (**Figure 3a**) to eliminate background interference and accurately define the ROI of each individual seed. Through comparative analysis of multi-band grayscale images, the band at 656.1300 nm presented the most significant contrast between seed targets and the black background, which was selected to construct a binary mask (**Figures 3b, c**). Based on preliminary experimental validation, an optimal segmentation threshold of 0.4 was determined. Pixels with gray values lower than 0.4 were defined as the background (assigned to 0), whereas pixels greater than or equal to 0.4 were defined as seed target regions (assigned to 1).

**Figure 3:**
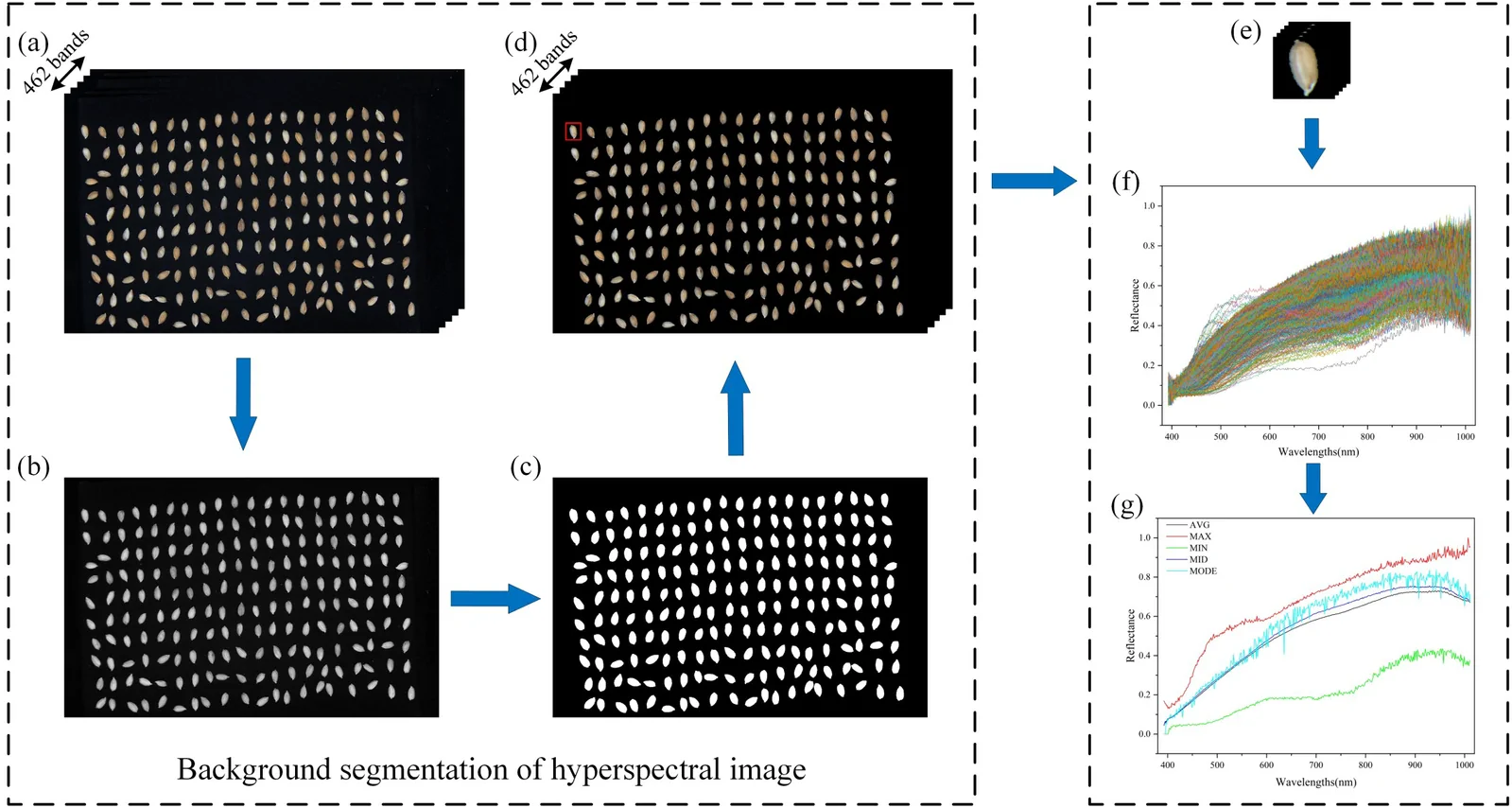
Workflow of spectral data extraction. **(a)** Calibrated original hyperspectral image; **(b)** grayscale image at 656.1300 nm with prominent contrast between rice seeds and the background; **(c)** binary mask image; **(d)** background-removed hyperspectral image; **(e)** region of interest (ROI) defined for each individual seed; **(f)** pixel-wise spectra extracted within the ROI; **(g)** five statistical spectral features (mean, maximum, minimum, median, and mode spectra) extracted from the ROI.

The generated binary mask was multiplied with all band layers of the original hyperspectral images to completely remove background noise and retain only valid seed regions (**Figure 3d**). Subsequently, individual seed images were segmented separately, and single-seed hyperspectral cubes with an average size of 50 × 26 × 462 were obtained (**Figure 3e**). To further verify the superiority of mean spectral features for seed identification, five statistical spectral features, including mean, maximum, minimum, median, and mid-value spectra within each seed ROI, were simultaneously extracted for comparative evaluation (**Figures 3f, g**). The multi-type spectral feature comparison provides a reliable data basis for subsequent optimal feature selection and model construction.

**Figures 4** and **5** illustrate the average spectral curves of the nine closely related japonica rice seed varieties within the spectral range of 392.38–1011.01 nm. All varieties exhibited highly consistent spectral variation trends and similar spectral response characteristics across the visible and near-infrared regions, with no distinguishable macroscopic differences. This phenomenon strongly demonstrates the high similarity in morphological and physicochemical properties among the tested japonica rice varieties, further verifying that traditional visual observation and conventional image-based methods are insufficient for accurate variety discrimination. In addition, the averaged spectral curves present high smoothness and low noise interference, which can reliably reflect the inherent holistic spectral characteristics of different rice seeds. Notably, subtle reflectance differences among varieties were observed in specific spectral bands. These fine spectral differences provide critical feature foundations and effective data support for the subsequent accurate classification and identification of closely related japonica rice varieties.

**Figure 4:**
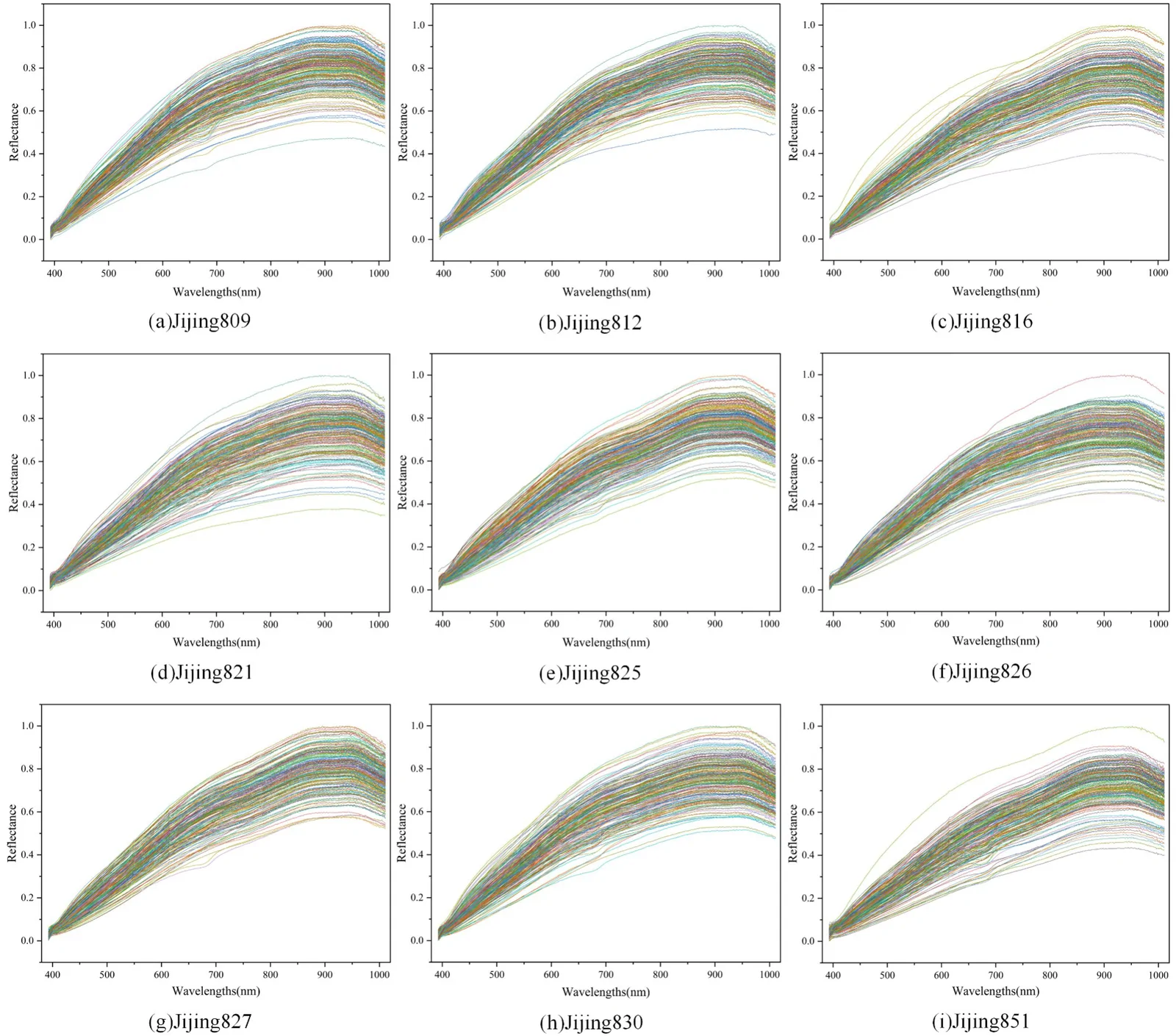
Average spectral profiles of all seeds in each category. Subplots: **(a)** Jijing809, **(b)** Jijing812, **(c)** Jijing816, **(d)** Jijing821, **(e)** Jijing825, **(f)** Jijing826, **(g)** Jijing827, **(h)** Jijing830, **(i)** Jijing851.

**Figure 5:**
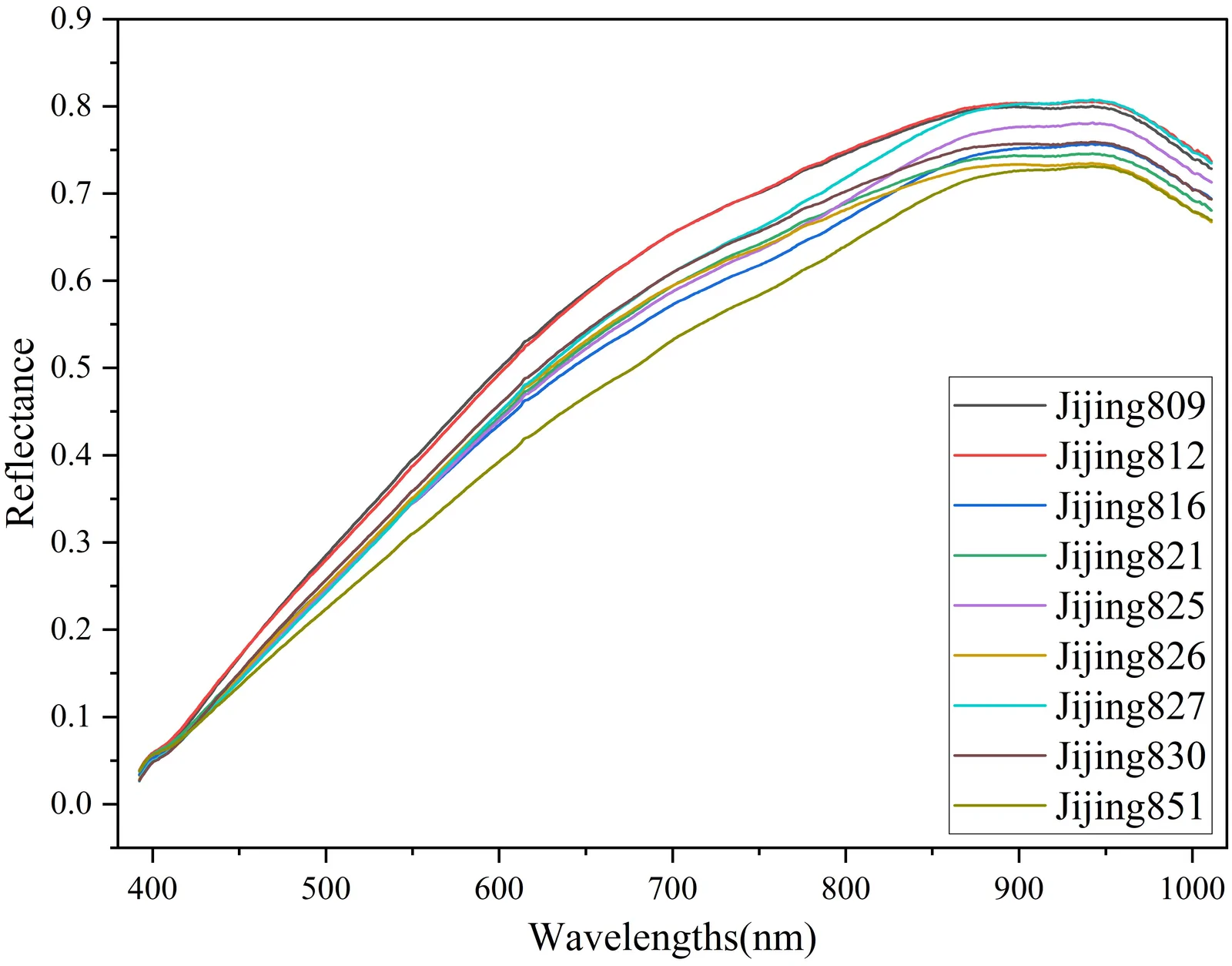
Comparison of average spectra for each category.

### 2.2 Model building

#### 2.2.1 Savitzky-Golay smoothing algorithm

Although the seed mean spectral data obtained by multi-pixel averaging can suppress partial random noise, the raw spectral curves still contain subtle high-frequency noise and burr interference caused by instrument precision limitations, ambient illumination fluctuations, and tiny morphological differences on seed surfaces. Such noise may mask genuine spectral differences and degrade the classification accuracy of subsequent models. Therefore, the Savitzky–Golay (SG) polynomial smoothing algorithm was employed in this study to preprocess the original mean spectra and eliminate high-frequency noise.

The SG algorithm is a time-domain smoothing and denoising method based on local polynomial least-squares fitting. Unlike conventional mean filtering and median filtering, the SG algorithm can effectively remove high-frequency random noise while maximally preserving spectral peak magnitudes, peak widths, and overall spectral trends. It avoids spectral distortion and feature offset, making it highly suitable for retaining subtle spectral variations in hyperspectral data. Owing to these advantages, SG smoothing has been widely applied in the spectral preprocessing of agricultural products.

The core principle of the SG algorithm is to perform high-order polynomial fitting on discrete spectral points within a fixed-size sliding window based on the least-squares criterion. The original spectral values are replaced by the optimal fitted values, thereby eliminating high-frequency noise while maintaining inherent spectral trends. The calculation formula is defined as Equation 2:

(2) $$y_{i}=\sum_{k=0}^{m}a_{k}{\left(i\right)}^{k}$$

where *y_i_* represents the fitted spectral value at the i-th wavelength within the sliding window, m is the polynomial order, and a denotes the polynomial fitting coefficients solved by minimizing the least-squares error of all sampling points within the window.

Through comparative parameter experiments, the optimal SG parameters were determined as a window size of 5 and a second-order polynomial. A relatively small window size prevents over-smoothing and preserves subtle spectral features, while the second-order polynomial accurately adapts to the gentle variation characteristics of seed spectral curves, balancing noise suppression and feature integrity.

After SG preprocessing, the high-frequency burrs and random noise in the spectral curves of different japonica rice varieties were effectively eliminated. The continuity and smoothness of the spectral curves were significantly improved, and the subtle spectral differences among closely related varieties were further highlighted. The signal-to-noise ratio and data reliability were greatly enhanced, providing high-quality spectral datasets for subsequent feature selection and stable model construction.

#### 2.2.2 Max-min normalization

Although the spectral data smoothed by the SG algorithm exhibit high stability, the reflectance values of different spectral bands present inconsistent numerical scales and overall spectral offsets caused by the physical properties of seed samples. These discrepancies may result in slow model convergence, imbalanced weight distribution, and degraded generalization performance during model training. To unify spectral dimensional scales and eliminate numerical differences across bands, Min-Max Normalization (MMN) was adopted for linear standardization of the denoised full-band spectral data. All spectral reflectance values were linearly mapped to the interval of [0, 1]. This method standardizes spectral data while retaining the relative variation characteristics of original spectral curves, thereby improving the training efficiency and classification accuracy of subsequent identification models. The MMN calculation formula is presented in Equation 3:

(3) $$x_{new}=\frac{x-x_{min}}{x_{max}-x_{min}}$$

where x represents the normalized spectral reflectance, x denotes the original reflectance value of a single band, and x and x are the maximum and minimum reflectance values across all spectral bands for an individual seed sample, respectively.

After MMN preprocessing, all spectral samples were constrained within a unified numerical range. The overall spectral offsets caused by illumination intensity, seed grain size, and surface flatness were effectively eliminated. Furthermore, the subtle spectral differences among closely related japonica rice varieties were well preserved, providing standardized and highly consistent input data for the construction of high-precision seed classification models.

#### 2.2.3 SPA feature selection algorithm

The original hyperspectral data employed in this study contain a total of 462 spectral bands, which suffer from high dimensionality, excessive redundant information, and severe multicollinearity among adjacent bands. Direct utilization of full-band spectral data for model training substantially increases computational complexity, reduces training convergence efficiency, and easily causes the curse of dimensionality, thereby inducing model overfitting and deteriorating generalization performance. To address these issues, the Successive Projections Algorithm (SPA) was adopted to screen optimal feature bands from the normalized high-dimensional spectral data, aiming to reduce data dimensionality, eliminate redundant variables, and enhance discriminative spectral features.

SPA is a forward iterative variable selection algorithm. Its core principle is to iteratively select feature bands with the minimum collinearity and lowest redundancy through vector projection analysis, so as to construct an optimal feature band combination. This algorithm can effectively eliminate multicollinearity interference among spectral bands and retain the most discriminative information using the fewest feature variables. Compared with other commonly used dimensionality reduction methods, such as principal component analysis and genetic algorithms, SPA selects physically interpretable spectral bands with superior targeted discrimination ability, making it highly applicable to spectral feature optimization and classification modeling of agricultural products. The iterative projection calculation of SPA is defined in Equation 4:

(4) $$proj\left(x_{k}\right)=x_{k}-\left(x_{k}^{T}x_{j\left(n\right)}\right)x_{j\left(n\right)}{\left(x_{j\left(n\right)}^{T}x_{j\left(n\right)}\right)}^{-1}$$

where $proj\left(x_{k}\right)$ represents the projection value of the candidate band vector x in the orthogonal space, and x and x denote the candidate band vector and the previously selected optimal band vector, respectively. The band with the maximum projection norm is selected as the new optimal feature band in each iteration until the termination condition is satisfied.

In this study, the SPA iteration range was set from 5 to 40 according to the characteristics of the japonica rice seed classification task, and the optimal number of feature bands was determined based on cross-validation classification accuracy. Finally, a set of representative, low-redundancy, and highly discriminative spectral bands were screened out. The dimensionality of spectral data was greatly reduced while the core varietal discrimination features of closely related japonica rice seeds were well preserved. The simplified input structure effectively improves the computational efficiency and recognition stability of the subsequent classification model, providing a compact and efficient feature dataset for the rapid and non-destructive identification of rice seeds.

#### 2.2.4 XGBoost algorithm

To achieve high-precision automatic identification of nine closely related japonica rice seed varieties, an Extreme Gradient Boosting (XGBoost) algorithm was employed to construct the seed classification model. As an optimized ensemble learning algorithm developed based on Gradient Boosting Decision Tree (GBDT), XGBoost builds a robust high-accuracy classifier by iteratively stacking and weighted fusing multiple weak decision trees. It exhibits prominent advantages in computational efficiency, generalization ability, and fault tolerance, making it highly suitable for hyperspectral classification tasks characterized by small sample sizes, high dimensionality, and subtle inter-class differences. Currently, XGBoost has been widely applied in the non-destructive identification and quality detection of agricultural products.

Different from conventional gradient boosting frameworks, XGBoost adopts a second-order Taylor expansion for loss function approximation to achieve more accurate gradient fitting of real errors. Meanwhile, regularization terms are integrated into the objective function to effectively alleviate overfitting. Compared with traditional machine learning models such as support vector machine and random forest, XGBoost presents superior stability and accuracy in fine feature discrimination and complex sample classification. The objective function consists of a training loss term and a regularization term, as defined in Equation 5:

(5) $$Obj=\sum_{i=1}^{n}l\left(y_{i},{\hat{y}}_{i}\right)+\sum_{k=1}^{K}\Omega \left(f_{k}\right)$$

where $l\left(y_{i},{\hat{y}}_{i}\right)$ represents the training loss function that quantifies the error between predicted and true labels; Ω(*f_k_*) denotes the regularization term containing L1 and L2 constraints to control the complexity of individual decision trees and prevent overfitting; K indicates the total number of decision trees.

During the iterative training process, the objective function was continuously minimized to optimize tree-splitting rules and weight parameters, enabling the model to automatically mine implicit spectral differences and feature correlations among closely related rice seeds. Finally, the optimized XGBoost model realized high-precision variety classification. Benefiting from the capability of capturing subtle spectral discrepancies from high-quality preprocessed data, the proposed model satisfies the requirements of rapid, accurate, and non-destructive identification of japonica rice seeds.

#### 2.2.5 Harris Hawks optimization

The performance of the conventional XGBoost model heavily relies on manually defined hyperparameters or grid search strategies. Such tuning approaches are highly subjective and tend to fall into local optimal solutions during the classification of closely related japonica rice seeds with subtle spectral differences, thereby limiting model generalization and restricting further improvements in classification accuracy. To overcome the drawbacks of manual parameter tuning and fully explore fine-grained spectral discrepancies among varieties, this study adopted Harris Hawks Optimization (HHO) to achieve global adaptive hyperparameter optimization for XGBoost. An optimized HHO-XGBoost model was finally constructed to improve both stability and identification accuracy for closely related japonica rice seed recognition.

HHO is a novel bionic meta-heuristic optimization algorithm that simulates the cooperative hunting, tracking, and sudden attacking behaviors of Harris hawks in nature. The algorithm adaptively switches between global exploration and local exploitation through dynamic prey escape energy regulation. Compared with traditional optimization algorithms such as genetic algorithm and particle swarm optimization, HHO exhibits distinct advantages, including fewer adjustable parameters, faster convergence speed, stronger global searching capability, and effective avoidance of premature convergence, which make it highly suitable for hyperparameter optimization in high-dimensional spectral modeling tasks.

(1) Prey escape energy update

The dynamic balance between global exploration and local exploitation is primarily controlled by prey escape energy, which serves as the core iterative regulation mechanism of HHO. The energy calculation is defined in Equation 6:

(6) $$E=2E_{0}\left(1-\frac{t}{T}\right)$$

where E represents the real-time prey escape energy, E is the initial random energy ranging from −1 to 1, t denotes the current iteration number, and T is the maximum iteration number. The algorithm performs global exploration when |E| ≥ 1 and switches to intensive local exploitation when |E|< 1.

(2) Population average position calculation

During local iterative optimization, the average population position is calculated to correct individual searching trajectories, as formulated in Equation 7:

(7) $$X_{m}\left(t\right)=\frac{1}{N}\sum_{i=1}^{N}X_{i}\left(t\right)$$

where X(t) is the average population position at the t-th iteration, N represents the total number of population individuals, and X(t) refers to the current position of the i-th individual.

(3) Global exploration position updating

In the global exploration stage, the algorithm searches the parameter space extensively and updates population positions through two random strategies, as shown in Equation 8:

(8) $$X\left(t+1\right)=\{\begin{array}{cc} X_{rand}\left(t\right)-r_{1}\left|X_{rand}\left(t\right)-2r_{2}X\left(t\right)\right| & q\geq 0.5\\ \left(X_{rabbit}\left(t\right)-X_{m}\left(t\right)\right)-r_{3}\left(L+r_{4}\left(U-L\right)\right) & q<0.5 \end{array}$$

where $X_{rand}\left(t\right)$ is the position of a randomly selected individual, $X_{rabbit}\left(t\right)$ represents the current optimal position, X(t) denotes the average population position, r1, r2, r3, r4, and q are uniformly distributed random values within [0, 1], and U and L correspond to the upper and lower boundaries of the parameter searching space, respectively.

(4) Four local exploitation strategies

In the local exploitation stage, HHO adaptively switches among four predation strategies according to the random factor r and the escape energy E to achieve fine-grained neighborhood searching. The four independent strategies cover all local optimization scenarios and are described as follows.

Hard besiege strategy (r ≥ 0.5, |E|< 0.5): The prey possesses low escape capability, and the hawk population conducts close-range rigid encirclement. The position update formula is expressed as Equation 9:

(9) $$X\left(t+1\right)=X_{rabbit}\left(t\right)-E\left|X_{rabbit}\left(t\right)-X\left(t\right)\right|$$

Soft besiege strategy (r ≥ 0.5, |E| ≥ 0.5): The prey maintains sufficient energy and continuous escape motion, and the hawks approach the optimal solution through flexible surrounding behavior. The position update formula is defined in Equation 10:

(10) $$X\left(t+1\right)=X_{rabbit}\left(t\right)-E\left|X_{rabbit}\left(t\right)-X_{m}\left(t\right)\right|$$

Progressive fast soft besiege (r< 0.5, |E| ≥ 0.5): The prey continuously jumps and escapes, and the hawks track dynamic jumping distances for adaptive searching. The position update formula is shown in Equation 11:

(11) $$X\left(t+1\right)=X_{rabbit}\left(t\right)-E\left|JX_{rabbit}\left(t\right)-X\left(t\right)\right|,\;J=2\left(1-r_{5}\right)$$

Progressive fast hard besiege (r< 0.5, |E|< 0.5): The prey exhausts escape energy with extremely weak mobility, enabling the hawk population to rapidly converge toward the optimal position. The position update formula is presented in Equation 12:

(12) $$X\left(t+1\right)=X_{rabbit}\left(t\right)-E\left|JX_{rabbit}\left(t\right)-X_{m}\left(t\right)\right|,\;J=2\left(1-r_{6}\right)$$

Overall, the HHO algorithm enables adaptive and global hyperparameter optimization of the XGBoost model, effectively solving the limitations of traditional empirical tuning and local optimal stagnation. The constructed HHO-XGBoost model possesses superior fitting ability and stability, which is conducive to achieving accurate classification of visually and genetically similar japonica rice seed varieties based on subtle hyperspectral features.

### 2.3 Model evaluation framework

Before implementing spectral preprocessing, feature screening and model optimization, raw hyperspectral data were divided via stratified random sampling at a 6:2:2 ratio into training, validation, and independent test sets. This stratified partitioning strategy ensured a consistent proportion of each japonica rice variety across all three datasets. To thoroughly eliminate cross-set information leakage, all data transformation and modeling fitting procedures were strictly limited to the training set. Specifically, the maximum and minimum spectral reflectance values for Min–Max normalization were exclusively calculated from training data and subsequently adopted to standardize validation and test samples. The Successive Projections Algorithm (SPA) for sensitive band selection was performed on the training set with 5-fold cross-validation, and the screened optimal spectral bands were directly extracted from validation and test datasets without secondary fitting. In addition, the Harris Hawks Optimization (HHO) algorithm tuned XGBoost hyperparameters by taking the training set’s 5-fold cross-validation accuracy as the fitness function. Throughout the entire experimental optimization process, the validation set was only used for intermediate model performance comparison, while the independent test set was completely isolated and only utilized to obtain final unbiased classification evaluation metrics.

To quantitatively and objectively evaluate the identification performance of the proposed model for nine highly similar japonica rice seed varieties, a comprehensive multi-dimensional evaluation system was established to systematically assess model accuracy, stability, and generalization capability. To avoid evaluation bias caused by a single metric, multiple indicators, including Accuracy, Precision, Recall, F1-Score, and Kappa coefficient, combined with a confusion matrix, were adopted for the multi-classification task. Considering the balanced sample distribution and the requirement of fair evaluation for highly similar germplasms, all metrics were calculated based on macro-average strategy (Trigka and Dritsas, 2025). This unbiased evaluation method equally quantifies the classification performance of each rice variety and eliminates evaluation distortion induced by sample size differences, thereby ensuring scientific and credible evaluation results.

As an intuitive visualization tool, the confusion matrix was employed to reveal the misclassification and omission distribution between true labels and predicted labels among nine japonica rice varieties. Four basic evaluation parameters were defined based on the confusion matrix: True Positive (TP) represents correctly predicted positive samples; True Negative (TN) denotes correctly discriminated negative samples; False Positive (FP) refers to negative samples incorrectly predicted as positive; and False Negative (FN) indicates positive samples misclassified as negative.

Precision (P) reflects the proportion of correctly classified samples within all predicted positive results, which evaluates the identification accuracy and misclassification level of the model for different japonica rice seeds. The calculation formula is shown in Equation 13:

(13) $$Precision=\frac{TP}{TP+FP}$$

Recall (R) represents the proportion of correctly predicted samples among all true positive samples, which quantifies the detection capability and omission error of the model. The mathematical expression is defined in Equation 14:

(14) $$Recall=\frac{TP}{TP+FN}$$

F1-Score is the harmonic mean of Precision and Recall, which comprehensively balances precision and recall performance and avoids one-sided evaluation caused by individual metric preference. It serves as a core indicator for assessing the overall classification performance. A value closer to 1 indicates superior comprehensive model performance, as calculated in Equation 15:

(15) $$F1=2\times \frac{Precision\times Recall}{Precision+Recall}$$

Accuracy (Acc) is the fundamental overall evaluation metric, representing the proportion of correctly predicted samples in the total test dataset. It intuitively reflects the overall identification ability of the model for nine closely related japonica rice seed varieties, as formulated in Equation 16:

(16) $$Accuracy=\frac{TP+TN}{TP+TN+FP+FN}$$

The Kappa coefficient was introduced to evaluate the consistency between predicted categories and ground-truth labels while eliminating the interference of random prediction errors and sample distribution imbalance. Its value ranges from 0 to 1, and a Kappa value closer to 1 indicates higher classification consistency and lower misclassification error. The calculation formula is presented in Equation 17:

(17) $$Kappa=\frac{P_{o}-P_{e}}{1-P_{e}}$$

where *P_o_* denotes the overall classification accuracy, and *P_e_* represents the expected consistency probability of random classification, which is used to eliminate evaluation deviation caused by unbalanced sample distribution.

In this study, macro-average calculation was adopted for Precision, Recall, and F1-Score to adapt to the nine-class classification task. The macro-average method calculates independent metric values for each variety and then obtains the arithmetic mean across all categories without sample weight bias. This strategy can objectively and fairly reflect the model’s ability to distinguish subtle spectral features of highly similar japonica rice seeds, ensuring comprehensive and reliable model evaluation.

### 2.4 Experimental environment and parameter settings

All data preprocessing, feature screening, model training and hyperparameter tuning experiments in this study were implemented on a desktop computer equipped with an Intel Core Ultra 7 270K Plus processor and 32 GB dual-channel DDR5 RAM (16 GB × 2, 8000 MHz, C36). The stable computing performance of this hardware configuration fully supports high-dimensional spectral matrix operations and iterative optimization, ensuring reliable experimental efficiency and result repeatability. All experiments were conducted on the Windows 11 operating system using Python 3.9 in PyCharm 2024. Specifically, the numpy and pandas libraries were employed for spectral data processing and matrix calculation; scipy was used to perform SG smoothing and Min–Max normalization; scikit-learn undertook dataset partitioning, SPA feature selection, evaluation metric calculation and confusion matrix visualization; XGBoost was adopted to construct the multi-class classification model, while matplotlib and seaborn were utilized for generating all experimental visualization graphs. To guarantee stable and reproducible modeling results, all experimental parameters were finalized through repeated pre-experiments. For spectral denoising, SG smoothing was performed with a window size of 5 and second-order polynomial fitting, which eliminated random noise while retaining subtle spectral differences among rice varieties. The SPA algorithm selected optimal sensitive bands ranging from 5 to 40 dimensions based on five-fold cross-validation accuracy, effectively removing collinear redundant features from the original 462-dimensional spectral data. For XGBoost modeling, hyperparameters including learning rate (0.01–0.3), maximum tree depth (2–10) and base learner number (50–200) were tuned, and L2 regularization was enabled to alleviate model overfitting. Meanwhile, the HHO optimizer was configured with a population size of 30 and a maximum of 50 iterations. It took the macro-average accuracy of the validation set as the fitness function to balance global exploration and local exploitation, thereby searching for the optimal hyperparameter combination for the identification of genetically similar japonica rice seeds.

## 3 Results and analysis

### 3.1 Original spectral analysis

**Table 1** shows XGBoost classification results of five pixel-level spectral features on raw hyperspectral data. All metrics are calculated as mean ± standard deviation from five repeated stratified dataset splits. Friedman test revealed statistically significant performance gaps between feature types (p< 0.01). The mean spectrum obtained the highest average accuracy (76.39 ± 0.0044, Kappa = 0.7315 ± 0.0054), significantly outperforming other spectral strategies (p< 0.05). Pixel averaging reduces noise from lighting and sensors and produces reliable discriminative features. The median spectrum ranked second (72.16 ± 0.0049), while mode, maximum and minimum spectra suffered severe noise interference with lower average accuracies of 70.43 ± 0.0052, 67.25 ± 0.0061 and 65.12 ± 0.0065, leading to frequent misclassification. Thus, mean spectra were adopted for follow-up modeling.

**Table 1:** Classification performance of XGBoost under different spectral extraction strategies.

Spectral type	Acc	P	R	F1	Kappa
Mode Spectrum	0.7043 ± 0.0052	0.7001 ± 0.0048	0.6972 ± 0.0055	0.6986 ± 0.0051	0.6682 ± 0.0063
Median Spectrum	0.7216 ± 0.0049	0.7173 ± 0.0050	0.7142 ± 0.0047	0.7157 ± 0.0048	0.6865 ± 0.0059
Max Spectrum	0.6725 ± 0.0061	0.6684 ± 0.0058	0.6657 ± 0.0062	0.6670 ± 0.0060	0.6341 ± 0.0071
Min Spectrum	0.6512 ± 0.0065	0.6474 ± 0.0063	0.6446 ± 0.0067	0.6460 ± 0.0065	0.6117 ± 0.0076
Mean Spectrum	0.7639 ± 0.0044	0.7582 ± 0.0041	0.7547 ± 0.0045	0.7564 ± 0.0043	0.7315 ± 0.0054

**Table 2** summarizes the classification performance of several conventional machine learning methods and a classic one-dimensional convolutional neural network (1D-CNN) baseline, which were both trained directly on raw mean spectral data. All quantitative indicators are reported as mean ± standard deviation, obtained from multiple independent random sampling experiments. The Friedman test was applied to evaluate statistical differences across model performances, and the results revealed extremely significant differences among all compared methods (p< 0.01).

**Table 2:** Comparison of classification performance among different machine learning models.

Machine learning model	Acc	P	R	F1	Kappa
Decision Tree (DT)	0.6125 ± 0.0072	0.6084 ± 0.0075	0.6053 ± 0.0078	0.6068 ± 0.0076	0.5716 ± 0.0083
Support Vector Machine (SVM)	0.6638 ± 0.0066	0.6597 ± 0.0069	0.6566 ± 0.0071	0.6581 ± 0.0070	0.6252 ± 0.0077
Logistic Regression (LR)	0.6342 ± 0.0069	0.6301 ± 0.0071	0.6270 ± 0.0074	0.6285 ± 0.0072	0.5943 ± 0.0080
K-Nearest Neighbor (KNN)	0.6476 ± 0.0067	0.6435 ± 0.0070	0.6404 ± 0.0072	0.6419 ± 0.0071	0.6082 ± 0.0079
Extra-Trees	0.7246 ± 0.0051	0.7205 ± 0.0053	0.7174 ± 0.0055	0.7189 ± 0.0054	0.6887 ± 0.0062
Random Forest (RF)	0.7015 ± 0.0055	0.6974 ± 0.0057	0.6943 ± 0.0059	0.6958 ± 0.0058	0.6641 ± 0.0066
1D-CNN	0.7308 ± 0.0058	0.7271 ± 0.0060	0.7243 ± 0.0062	0.7257 ± 0.0061	0.6954 ± 0.0068
XGBoost	0.7639 ± 0.0044	0.7582 ± 0.0041	0.7547 ± 0.0045	0.7564 ± 0.0043	0.7315 ± 0.0054

Single classifiers, namely DT, LR and KNN, exhibited unsatisfactory recognition ability, with average accuracy values ranging only from 0.6125 ± 0.0072 to 0.6476 ± 0.0067. Limited by insufficient nonlinear fitting capability and severe redundancy of high-dimensional spectral data, SVM also achieved a low accuracy of 0.6638 ± 0.0066. As typical parallel ensemble models, RF and Extra-Trees outperformed individual single classifiers, yielding average accuracies of 0.7015 ± 0.0055 and 0.7246 ± 0.0051, respectively. Nevertheless, these two algorithms lack iterative error correction strategies, making it difficult to capture subtle spectral differences among similar rice varieties.

The 1D-CNN model, a widely used deep learning architecture for sequential spectral signals, achieved a moderate classification accuracy of 0.7308 ± 0.0058. Despite its powerful automatic feature learning advantage, CNNs rely heavily on sufficient labeled samples to prevent overfitting. Restricted by the limited sample size of this nine-class rice seed classification task, the 1D-CNN suffered unstable training convergence and could not effectively extract minute spectral discrepancies among genetically similar varieties, thereby showing poorer performance than XGBoost.

In comparison, XGBoost achieved the best overall performance, with an average accuracy of 0.7639 ± 0.0044 and an average Kappa coefficient of 0.7315 ± 0.0054. The *post-hoc* Nemenyi test further verified that XGBoost was statistically superior to 1D-CNN and all other competing models (p< 0.05). Owing to its second-order gradient loss optimization and inherent regularization constraints, XGBoost presents robust fitting ability on small-scale datasets. It can effectively capture subtle spectral differences of closely related japonica rice seeds while suppressing overfitting. Accordingly, XGBoost was determined as the base classifier for the subsequent multi-stage optimization experiments in this study.

### 3.2 Spectral denoising

**Figure 6** illustrates the identification performance of the XGBoost model adopting six preprocessing methods, namely raw spectrum, mean filtering, median filtering, Gaussian filtering, wavelet threshold denoising, and Savitzky–Golay smoothing. The raw spectral data without denoising achieved the poorest comprehensive performance with an accuracy of 76.39%, indicating that inherent spectral noise severely restricted the identification of different rice varieties.

**Figure 6:**
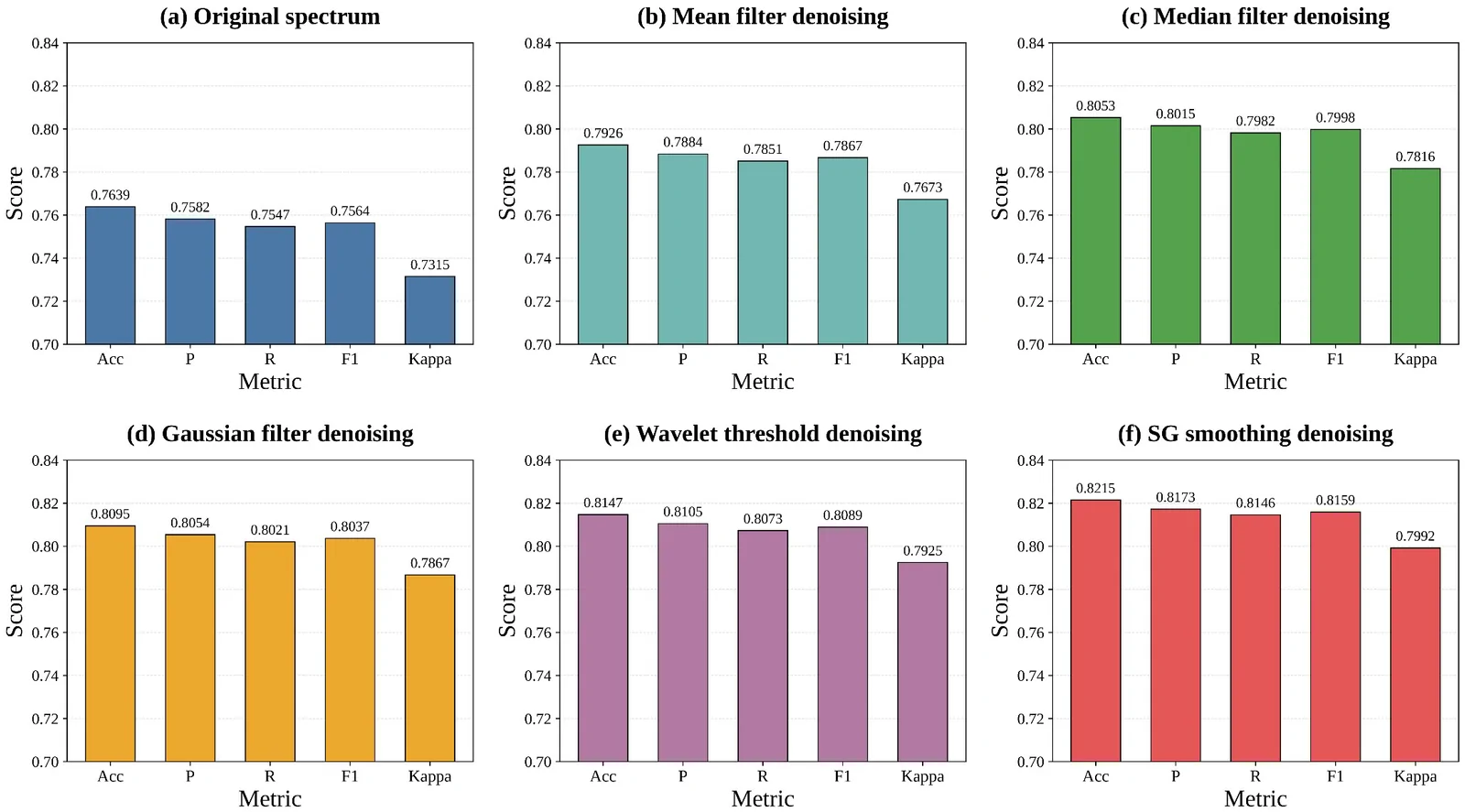
Comparison results of different noise reduction methods. Subplots: **(a)** Original spectrum, **(b)** Mean filter denoising, **(c)** Median filter denoising, **(d)** Gaussian filter denoising, **(e)** Wavelet threshold denoising, **(f)** SG smoothing denoising.

Mean filtering slightly improved the model accuracy to 79.26% by reducing partial noise, yet it tended to blur detailed spectral features and only produced limited performance gains. Median filtering and Gaussian filtering achieved better noise suppression for impulse noise and Gaussian random noise, yielding accuracies of 80.53% and 80.95%, respectively. Wavelet threshold denoising realized effective separation of valid spectral signals and noise in the time–frequency domain, further increasing the accuracy to 81.47% and outperforming the above three filtering methods.

Savitzky–Golay smoothing contributed the optimal model performance across all evaluation metrics. The model accuracy reached 82.15%. The macro-average precision, macro-average recall, and macro-average F1-score were 81.73%, 81.46%, and 81.59%, respectively, with a Kappa coefficient of 0.7992. This method removes high-frequency noise based on sliding least-squares polynomial fitting while completely retaining spectral absorption characteristics and subtle spectral differences among japonica rice varieties. Therefore, SG smoothing was selected as the optimal spectral denoising preprocessing method in this study.

### 3.3 Recognition performance after data normalization

**Figure 7a** presents the confusion matrix derived from spectral data processed merely by SG smoothing. The classification accuracy of the nine japonica rice varieties ranged from 80.0% to 84.6%. Both the 3rd and 5th varieties achieved the lowest accuracy of 80.0%, with their misclassified samples dispersedly distributed across multiple genetically similar varieties instead of being limited to adjacent categories. Most off-diagonal misclassification probabilities ranged between 2.5% and 5.0%, which reflected insufficient feature distinguishability among these closely related rice germplasms.

**Figure 7:**
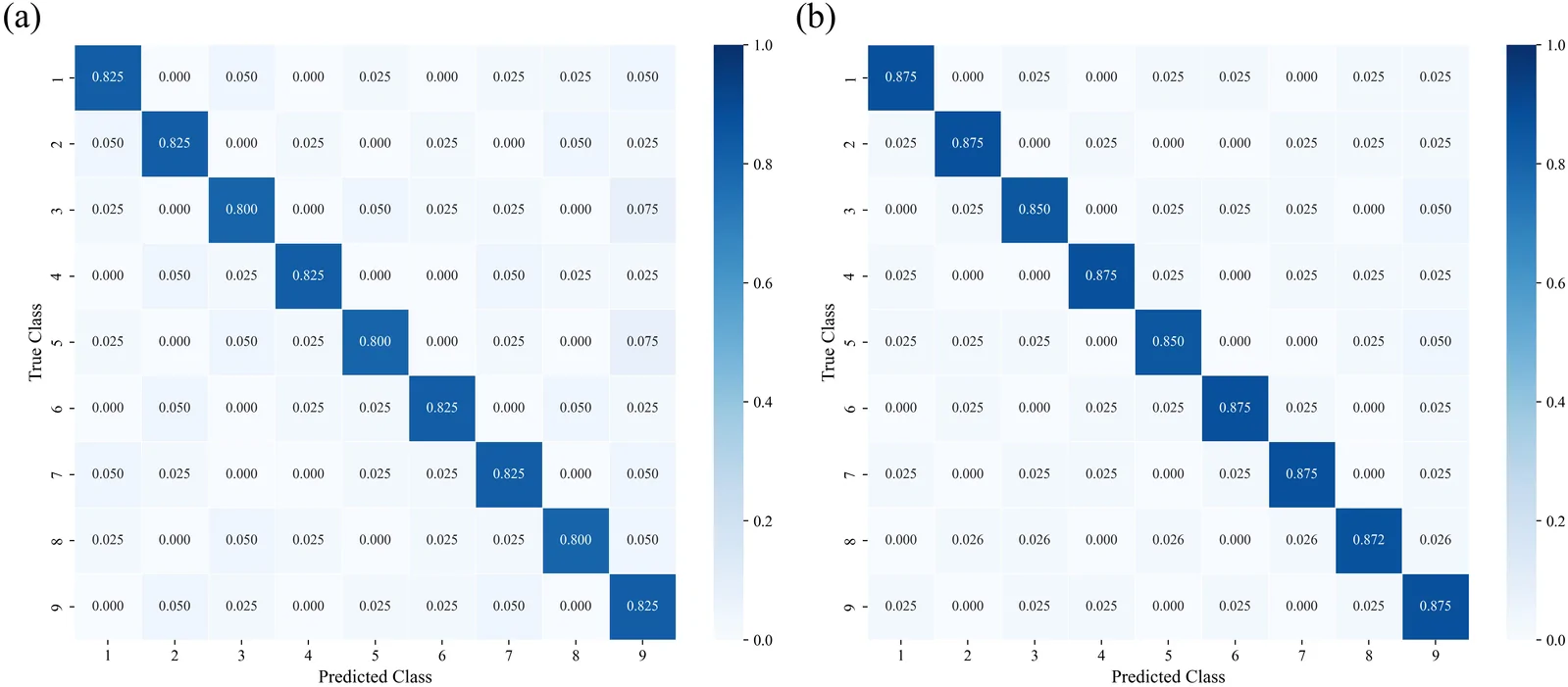
Comparison of confusion matrices before and after normalization. **(a)** Before normalization; **(b)** After normalization.

**Figure 7b** further shows the classification results after implementing MMN normalization on the SG-smoothed spectral data. The overall classification performance was significantly improved, with the accuracy of all varieties stably maintained at 85.0%–87.5%. By eliminating dimensional differences and spectral amplitude fluctuations, MMN normalization effectively corrected feature bias during model training. In particular, the accuracy of the easily confused 3rd and 5th varieties increased from 80.0% to 85.0%, while the overall misclassification rate decreased obviously. Most inter-class classification errors were reduced to approximately 2.5% after dual preprocessing.

This comparative verification indicates that MMN normalization can optimize the distribution characteristics of hyperspectral features and mitigate misclassification among genetically similar rice varieties. The combination of SG smoothing and MMN normalization achieves complementary optimization effects, jointly improving the classification accuracy and stability of the identification model for japonica rice seeds with low genetic differentiation.

### 3.4 Classification results after feature selection

**Table 3** summarizes the classification performance of seven feature optimization pipelines following SG smoothing and Min–Max normalization preprocessing. All evaluation metrics are reported as mean ± standard deviation calculated from repeated independent random sampling trials to quantify experimental random fluctuations. A Friedman test was adopted to examine inter-group performance discrepancies, and statistically significant differences were detected among all feature screening approaches (p< 0.01). The full original spectrum containing 462 wavelength bands delivered the lowest classification accuracy, which is mainly caused by severe spectral redundancy and multicollinearity across adjacent bands. Subsequent dimensionality reduction and feature screening strategies gradually improved model predictive performance. PCA compressed raw spectral data into 35 orthogonal principal components and yielded moderate classification results. LASSO regularization, random forest feature importance ranking, mutual information and grey relational analysis further shrank feature dimensions while continuously lifting overall classification accuracy. Among all comparative schemes, the SPA only retained 22 sensitive spectral bands and achieved the best comprehensive evaluation metrics within this group of experiments. By iteratively selecting wavelength combinations with low collinearity, SPA helps filter out redundant interference bands and reserve spectral variables correlated with japonica seed traits under this dataset; thus, SPA was adopted as the feature extraction strategy for subsequent modeling.

**Table 3:** Comparison of model classification performance under different feature processing methods.

Feature processing method	Feature dimension	Acc	P	R	F1	Kappa
Full-band spectrum	462	0.8628 ± 0.0055	0.8594 ± 0.0057	0.8567 ± 0.0059	0.8580 ± 0.0058	0.8476 ± 0.0064
Principal Component Analysis (PCA)	35	0.8847 ± 0.0050	0.8815 ± 0.0052	0.8792 ± 0.0053	0.8803 ± 0.0052	0.8721 ± 0.0058
LASSO Regularization Feature Selection	29	0.8962 ± 0.0048	0.8934 ± 0.0049	0.8907 ± 0.0051	0.8920 ± 0.0050	0.8853 ± 0.0055
Random Forest Feature Importance (RF)	27	0.9015 ± 0.0046	0.8986 ± 0.0047	0.8963 ± 0.0049	0.8974 ± 0.0048	0.8911 ± 0.0053
Mutual Information (MI)	25	0.9067 ± 0.0043	0.9039 ± 0.0044	0.9015 ± 0.0046	0.9027 ± 0.0045	0.8964 ± 0.0050
Grey Relational Analysis (GRA)	24	0.9092 ± 0.0042	0.9065 ± 0.0043	0.9041 ± 0.0044	0.9053 ± 0.0044	0.8991 ± 0.0048
Successive Projections Algorithm (SPA)	22	0.9153 ± 0.0040	0.9126 ± 0.0041	0.9102 ± 0.0042	0.9114 ± 0.0042	0.9042 ± 0.0046

This study employed SHAP analysis to explain the classification mechanism of the 22 SPA-selected sensitive bands and improve the limited interpretability of ensemble-based XGBoost model. The overall feature importance ranking in **Figure 8a** shows that the spectral bands at 368 nm, 231 nm, and 95 nm contributed most strongly to variety discrimination, with the remaining bands decreasing gradually in predictive weight, which quantitatively verifies the validity of the SPA feature screening results. The SHAP bee swarm plot in **Figure 8b** further reveals diverse feature response behaviors: several core bands presented clear positive and negative correlations with spectral reflectance, while most wavelengths exhibited mixed and complex response patterns. This complex feature variation is the primary reason for the difficulty in differentiating genetically similar japonica rice varieties with subtle spectral discrepancies. In addition, the sample-level SHAP heatmap in **Figure 8c** demonstrates that individual spectral bands exert inconsistent predictive effects across different seed samples, reflecting the adaptive characteristics of the screened feature subset. Overall, SHAP interpretation effectively resolves the black-box problem of the XGBoost model, provides reliable data-driven explanations for optimized spectral features, and lays a foundation for further exploring the physicochemical mechanisms corresponding to key sensitive bands of japonica rice seeds.

**Figure 8:**
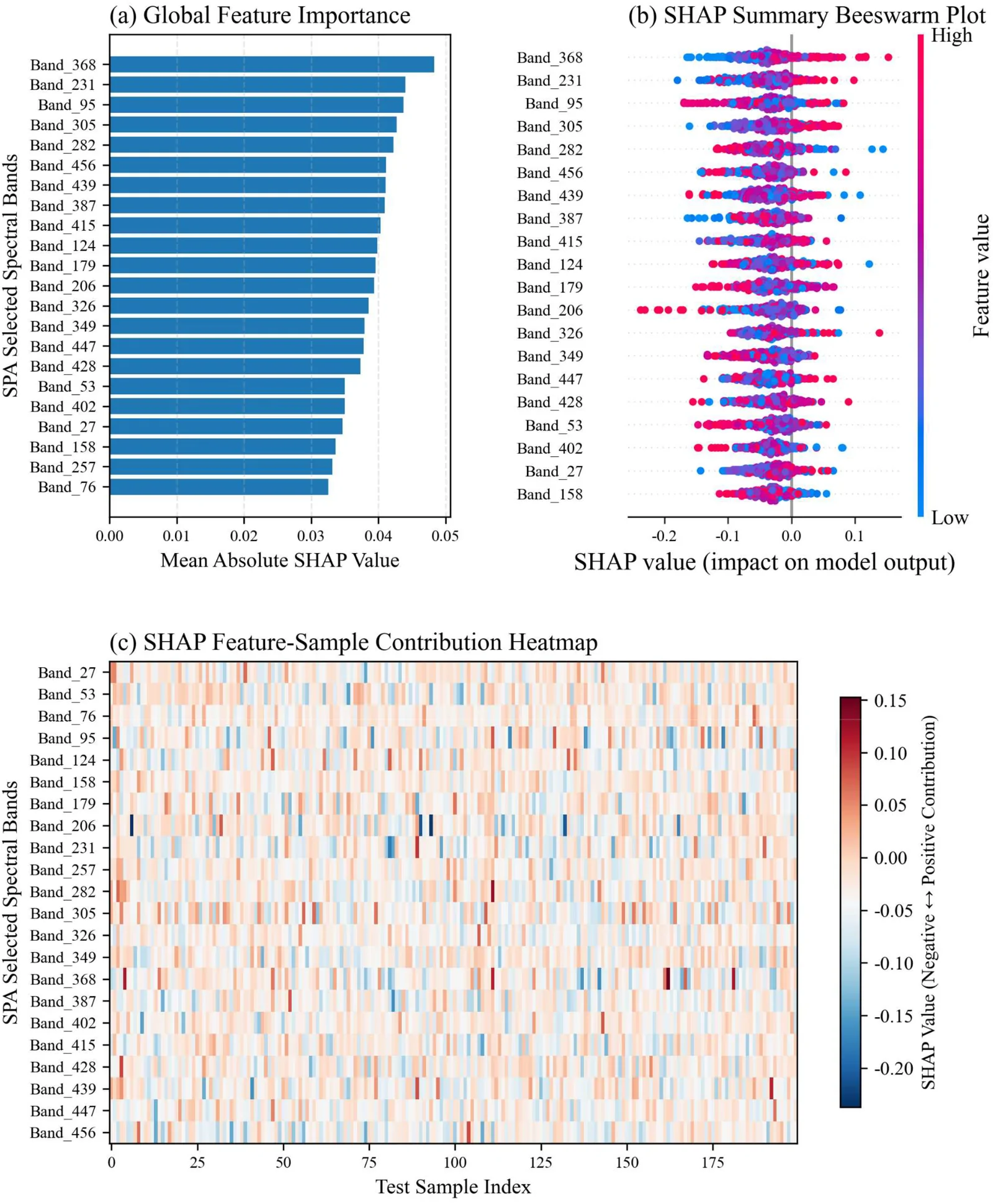
Analysis of feature importance selected by the SPA algorithm. **(a)** Global Feature Importance; **(b)** SHAP Summary Beeswarm Plot; **(c)** SHAP Feature-Sample Contribution Heatmap.

### 3.5 Results of the optimization using swarm intelligence algorithms

**Table 4** summarizes the quantitative indicators of XGBoost models optimized by nine swarm intelligence meta-heuristic algorithms, namely Particle Swarm Optimization (PSO), Grey Wolf Optimizer (GWO), Whale Optimization Algorithm (WOA), Genetic Algorithm (GA), Bat Algorithm (BA), Dragonfly Algorithm (DA), Ant Colony Optimization (ACO), Sparrow Search Algorithm (SSA), and Harris Hawks Optimization (HHO), based on SPA-screened spectral features. All evaluation metrics are presented in the form of mean ± standard deviation obtained from multiple independent random sampling trials to reflect experimental random errors. A Friedman test was adopted for overall inter-group comparison, and the result revealed statistically significant performance discrepancies among the nine optimization algorithms (p< 0.01).

**Table 4:** Comparison of the classification performance of XGBoost models optimized using different swarm intelligence algorithms.

Optimization methods	Optimal parameters (learning rate, maximum depth, number of base learners)	Acc	P	R	F1	Kappa
PSO-XGBoost	(0.086,7,142)	0.9346 ± 0.0042	0.9321 ± 0.0044	0.9298 ± 0.0046	0.9309 ± 0.0045	0.9250 ± 0.0051
GWO-XGBoost	(0.079,6,138)	0.9389 ± 0.0040	0.9364 ± 0.0041	0.9342 ± 0.0043	0.9353 ± 0.0042	0.9297 ± 0.0048
WOA-XGBoost	(0.077,6,139)	0.9403 ± 0.0039	0.9378 ± 0.0040	0.9356 ± 0.0042	0.9367 ± 0.0041	0.9312 ± 0.0047
GA-XGBoost	(0.082,7,144)	0.9365 ± 0.0041	0.9340 ± 0.0042	0.9317 ± 0.0044	0.9328 ± 0.0043	0.9269 ± 0.0049
BA-XGBoost	(0.073,6,137)	0.9446 ± 0.0036	0.9422 ± 0.0037	0.9399 ± 0.0039	0.9410 ± 0.0038	0.9358 ± 0.0044
DA-XGBoost	(0.076,7,140)	0.9414 ± 0.0038	0.9390 ± 0.0039	0.9367 ± 0.0041	0.9378 ± 0.0040	0.9324 ± 0.0046
ACO-XGBoost	(0.074,6,143)	0.9421 ± 0.0037	0.9397 ± 0.0038	0.9374 ± 0.0040	0.9385 ± 0.0039	0.9331 ± 0.0045
SSA-XGBoost	(0.075,6,141)	0.9425 ± 0.0037	0.9401 ± 0.0038	0.9378 ± 0.0040	0.9389 ± 0.0039	0.9336 ± 0.0045
HHO-XGBoost	(0.072,6,136)	0.9472 ± 0.0034	0.9448 ± 0.0035	0.9426 ± 0.0036	0.9437 ± 0.0036	0.9389 ± 0.0041

Among the nine hybrid models, PSO-XGBoost yielded the lowest classification performance, which is largely attributable to its tendency of premature convergence and trapping in local optima. Moderate classification accuracy was obtained by GWO-XGBoost, WOA-XGBoost, GA-XGBoost, DA-XGBoost and ACO-XGBoost, which are constrained by insufficient global search capability during hyperparameter tuning. BA-XGBoost and SSA-XGBoost achieved comparatively competitive prediction results. From the perspective of multiple quantitative evaluation indicators, the HHO-XGBoost model attained the maximum average Acc of 0.9472, as well as the optimal P, R, F1 and Kappa metrics within the comparison group.

A McNemar test was further performed for pairwise statistical comparison between PSO-XGBoost (the model with the worst comprehensive performance) and HHO-XGBoost. The test result demonstrated that the accuracy difference between the two models was statistically significant (p< 0.05). The HHO algorithm adopts an adaptive prey escape energy strategy to dynamically coordinate global exploration and local exploitation stages. This mechanism helps alleviate premature convergence risks and can identify suitable hyperparameter combinations for the classification task of genetically similar japonica rice seeds under the experimental dataset in this study.

**Figure 9** presents the iterative convergence curves of the nine swarm intelligence algorithms within 50 iterations, with XGBoost’s five-fold cross-validation accuracy defined as the fitness function. Slight differences in initial fitness values were attributed to random population initialization of each algorithm. All methods rapidly improved model performance in the early iteration stage via extensive hyperparameter searching. In detail, PSO converged prematurely at approximately the 20th iteration and finally stabilized at 93.46%, demonstrating limited global search capacity in this rice seed classification scenario. Although GWO and WOA effectively avoided early local convergence, they only achieved moderate final accuracy on the current dataset. By contrast, DA, ACO and SSA maintained continuous exploration of the hyperparameter space throughout the entire iterative process and showed steadily optimized convergence trends. Among all comparison algorithms, HHO sustained effective global exploration in the first 35 iterations and finally converged at the highest accuracy of 94.72%. Relying on its adaptive prey escape energy mechanism, HHO dynamically balances global exploration and local exploitation during optimization, which effectively prevents premature convergence and enables the acquisition of hyperparameter combinations suitable for fine-grained identification of genetically similar japonica rice seeds. Notably, the above results only reflect the relative performance of the nine meta-heuristic algorithms under the fixed spectral dataset and XGBoost framework established in this study, rather than proving the universal superiority of the HHO algorithm.

**Figure 9:**
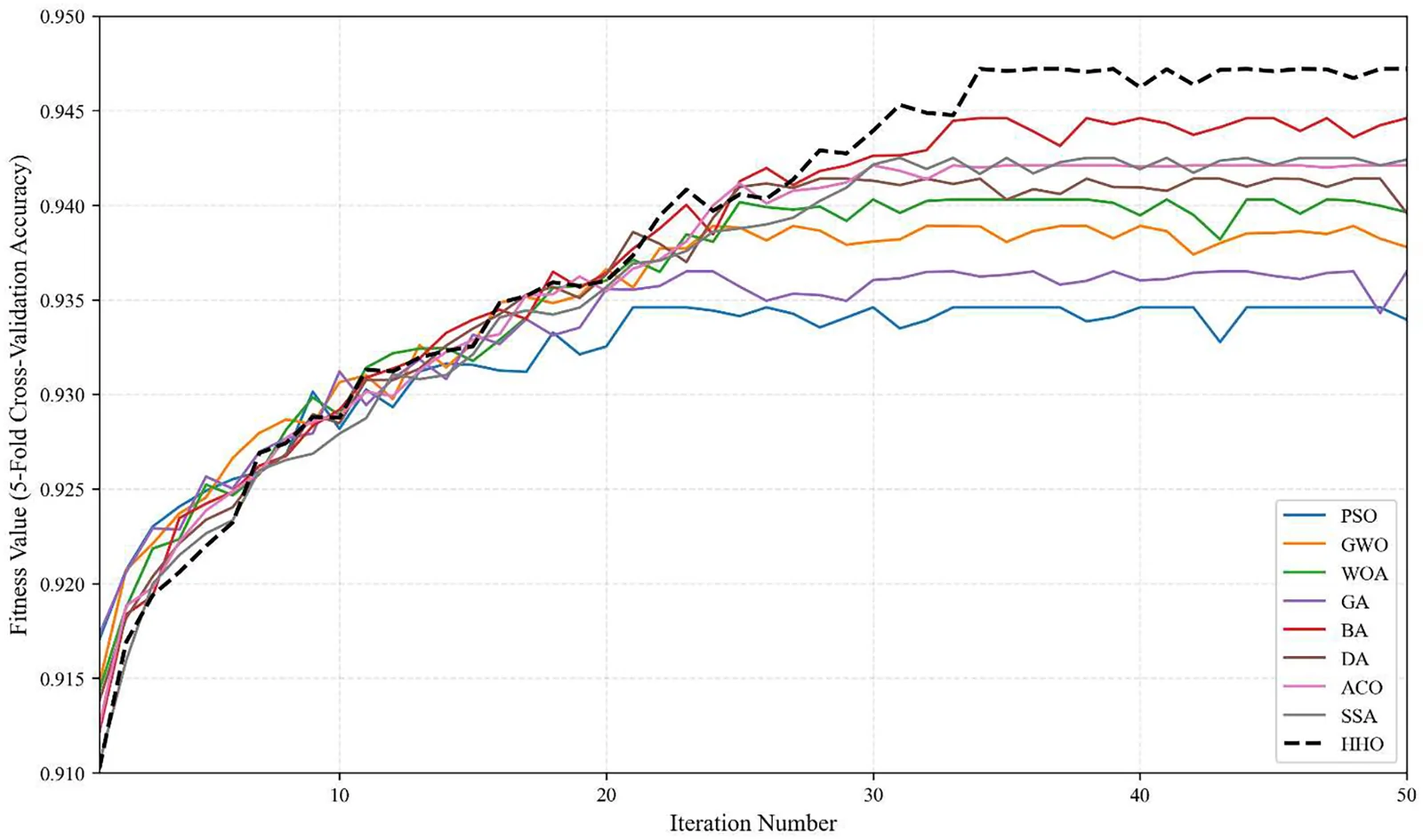
Convergence curves for nine swarm intelligence optimization algorithms. The fitness values correspond to the 5-fold cross-validation accuracy of the XGBoost model. The different initial fitness values result from the random initialization of the populations.

**Figure 10** shows the normalized confusion matrices of nine swarm intelligence-optimized XGBoost models for the classification of nine morphologically similar japonica rice varieties. The diagonal values represent the correct classification proportion of each variety, while off-diagonal entries correspond to inter-variety misclassification rates. All hybrid models achieved satisfactory overall recognition performance with most seed samples correctly identified, and misclassifications were dispersedly distributed across various genetically similar varieties rather than concentrated among adjacent categories. In particular, PSO-XGBoost and GA-XGBoost showed relatively poor discriminative ability for the 1st, 2nd and 3rd rice varieties, with random cross-category misjudgments occurring across non-adjacent labels. In general, algorithms with stronger global search capabilities yielded higher diagonal classification accuracy and lower overall misclassification rates in this comparison. Among all constructed models, HHO-XGBoost (**Figure 10i**) produced the most competitive classification results, maintaining the highest per-variety correct prediction proportion with only sporadic misclassified samples, which effectively reduced classification errors among genetically analogous rice germplasms within this experimental framework. Such results demonstrate that the HHO optimizer can effectively escape local optima, fully mine subtle discriminative spectral features, and further improve the classification accuracy and stability for highly similar japonica rice seeds. Nevertheless, this performance advantage is specific to the current dataset and model configuration and does not indicate the universal superiority of HHO over other meta-heuristic algorithms.

**Figure 10:**
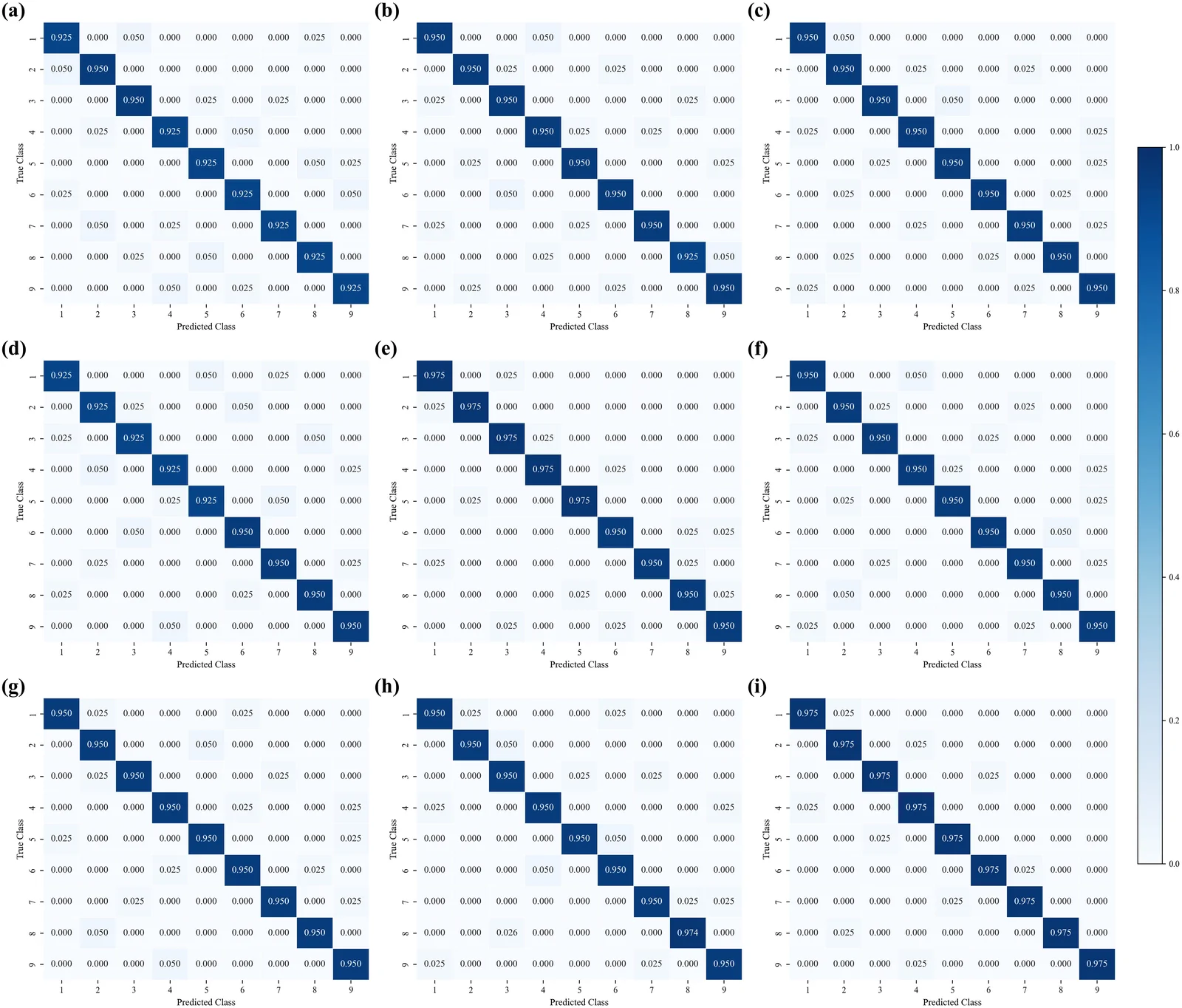
Confusion matrix for nine swarm intelligence optimization algorithms. **(a)** PSO; **(b)** GWO; **(c)** WOA; **(d)** GA; **(e)** BA; **(f)** DA; **(g)** ACO; **(h)** SSA; **(i)** HHO.

**Table 5** quantitatively illustrates the incremental performance improvements brought by individual spectral preprocessing and hyperparameter optimization modules. All evaluation metrics are presented as mean ± standard deviation, calculated from repeated independent random sampling experiments. The Friedman test was performed to compare the five progressively optimized modeling pipelines, and the results confirmed statistically significant differences in performance among all modeling schemes (p< 0.01).

**Table 5:** Step-by-step comparison of model classification performance under different preprocessing and optimization strategies.

Method	Acc	P	R	F1	Kappa
XGBoost	0.7639 ± 0.0044	0.7582 ± 0.0041	0.7547 ± 0.0045	0.7564 ± 0.0043	0.7315 ± 0.0054
XGBoost-SG	0.8215 ± 0.0041	0.8173 ± 0.0042	0.8146 ± 0.0043	0.8159 ± 0.0042	0.7992 ± 0.0049
XGBoost-SG-MMN	0.8628 ± 0.0055	0.8594 ± 0.0057	0.8567 ± 0.0059	0.8580 ± 0.0058	0.8476 ± 0.0064
XGBoost-SG-MMN-SPA	0.9153 ± 0.0040	0.9126 ± 0.0041	0.9102 ± 0.0042	0.9114 ± 0.0042	0.9042 ± 0.0046
XGBoost-SG-MMN-SPA-HHO	0.9472 ± 0.0034	0.9448 ± 0.0035	0.9426 ± 0.0036	0.9437 ± 0.0036	0.9389 ± 0.0041

The baseline XGBoost model constructed using raw spectral data yielded the poorest classification performance. This inferior performance stems from inherent defects of original hyperspectral data, including random spectral noise, inconsistent numerical magnitudes across different wavelength bands, and massive redundant spectral features. SG smoothing was firstly applied to eliminate high-frequency spectral burrs, which achieved a noticeable enhancement in average classification accuracy. On the basis of smoothed spectra, Min–Max normalization further eliminated numerical offsets among spectral bands and effectively boosted the overall classification capability of the model. Subsequent SPA feature selection removed collinear redundant wavelengths and refined core discriminative spectral features, producing a further obvious improvement in classification performance.

In the final optimization step of the progressive workflow, the HHO meta-heuristic algorithm was introduced to conduct adaptive hyperparameter tuning for XGBoost. Among the five constructed modeling pipelines, this fully optimized framework achieved the highest average values of accuracy, precision, recall, F1-score and Kappa coefficient. *Post-hoc* Nemenyi tests validated that each incremental processing step contributed a statistically significant performance gain (p< 0.05). This hierarchical multi-stage framework realizes step-by-step optimization by suppressing spectral noise, standardizing feature scales, filtering redundant variables, and optimizing model hyperparameters. The integrated strategy substantially strengthens the feature discriminative ability for genetically similar japonica rice seeds and ultimately achieves accurate and stable variety identification.

The F1-score, as the harmonic mean of precision and recall, avoids evaluation bias caused by unbalanced samples and objectively reflects the classification performance of individual rice varieties. **Figure 11a** presents the F1-score heatmap of nine rice categories under five models, illustrating the performance improvement brought by stepwise denoising, normalization, feature selection, and hyperparameter optimization. The baseline XGBoost model yielded low F1-scores across all categories. In particular, the third category obtained the lowest F1-score of 71.9%, with severe misclassification among similar varieties. With the sequential implementation of SG smoothing, MMN, SPA feature selection, and HHO optimization, the F1-scores of all categories increased steadily. The final optimized model achieved F1-scores above 92.9% for all nine categories, and the originally confused third, sixth, and eighth varieties obtained the most significant improvement, indicating enhanced inter-variety discriminability.

**Figure 11:**
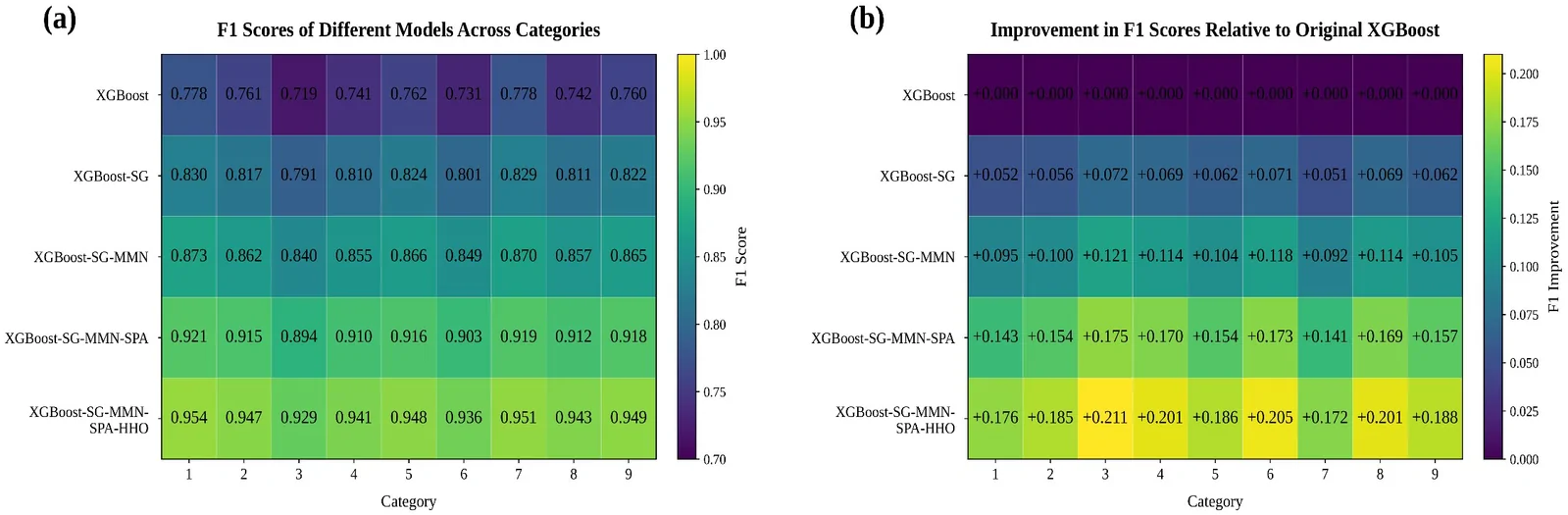
Comparison of F1 scores across categories during the model optimization process. **(a)** F1 Scores of Different Models Across Categories; **(b)** Improvement in F1 Scores Relative to Original XGBoost.

**Figure 11b** quantifies the F1-score improvement at each optimization stage relative to the baseline model. Savitzky–Golay smoothing improved F1-scores by 5.1% to 7.2%. Further Min-Max normalization expanded the improvement range to 9.2%–12.1%. SPA feature selection increased most category accuracies by more than 14.0%. After HHO hyperparameter optimization, the third, sixth, and eighth varieties achieved the top three improvements of 21.1%, 20.5%, and 20.1%, respectively. The results demonstrate that the proposed hierarchical optimization strategy not only enhances overall classification accuracy but also specifically corrects misjudgments of highly similar japonica rice varieties, which validates the effectiveness and robustness of the entire preprocessing and optimization framework.

## 4 Discussion

To achieve accurate, non-destructive identification of japonica rice seeds with high genetic similarity, we built an integrated analytical framework based on hyperspectral imaging. This workflow combines multi-step spectral preprocessing, optimal feature selection, SHAP-based interpretive analysis, and swarm-intelligence-driven hyperparameter tuning. Through systematic comparisons among multiple algorithms, our final HHO-XGBoost model achieved high classification accuracy across nine closely-related japonica rice varieties. This framework mitigates well-known drawbacks of raw hyperspectral data, including noise contamination and severe feature redundancy. It also reduces the risk of local optimal solutions commonly encountered during manual hyperparameter adjustment. SHAP outputs further pinpointed key sensitive spectral bands and clarified how these features contribute to variety discrimination. These findings offer practical technical references for high-throughput, non-destructive screening of genetically similar crop germplasm.

Even so, the complete XGBoost-SG-MMN-SPA-HHO pipeline demands relatively high computational overhead during model training. Repeated SPA feature selection together with HHO-based hyperparameter searching extends training runtime when compared with hardware-accelerated lightweight CNN architectures. Despite higher computational cost, the compact feature subset generated by SPA creates favorable conditions for subsequent model interpretation. Beyond dimensionality reduction, our workflow delivers strong interpretability, which opens opportunities to infer physiological and biochemical mechanisms behind spectral responses for germplasm identification. Lightweight CNN models, by contrast, benefit from fast hardware-assisted training. Yet they generally need larger sample volumes and behave as black-box models; their internal decision logic is hard to unpack, which limits our ability to explore underlying spectral mechanisms. Reducing computation costs for swarm-intelligence optimization will be prioritized in our follow-up research.

While our model yields promising classification performance under controlled laboratory settings, this study still suffers from several quantifiable limitations.

First, constraints exist regarding sample size and stratified sampling design. We applied strict stratified random splitting following a 6:2:2 ratio, ensuring that each japonica rice variety maintains identical sample proportions within training, validation and independent test partitions. This approach guarantees fair macro-level performance evaluation. Each variety includes 200 seed samples in total. After stratified division, roughly 120 samples per class enter the training set, 40 go to the validation set, and another 40 are reserved for independent testing. For this nine-class classification task, only 40 independent test samples are available for each rice variety. Published chemometric and agricultural spectroscopy studies suggest that at least 80–100 samples per class are preferred to produce stable macro-F1 metrics with low variance. With merely 40 test samples for each class, classification metrics inevitably show certain random fluctuations. This represents a concrete quantitative constraint rather than a vague qualitative shortcoming.

Second, all spectral measurements were collected under stable, standardized laboratory conditions. Real-world field interference factors, such as variable illumination, ambient temperature and humidity, seed moisture fluctuation, and seed aging during long-term storage, were not incorporated into our dataset. Since samples come from a single data distribution without multi-scenario disturbance inputs, model robustness may decline, and classification accuracy could drop sharply when deployed for on-site seed detection.

Third, experimental germplasm covers only nine japonica rice accessions collected from one research institute in Jilin Province and harvested within a single year. Such limited sample coverage restricts our model to closed-set classification scenarios. It cannot handle unknown, unlabeled rice materials or support large-scale seed resource screening across multiple regions.

Fourth, only one-dimensional spectral reflectance signals were used for model construction. Spatial texture, grain morphology and color information embedded within hyperspectral images were not fused into modelling inputs. Missing spatial features may hinder further performance improvement for rice varieties with extremely subtle spectral differences.

Fifth, the proposed algorithm pipeline currently runs only for offline computer analysis. We have not completed model compression, embedded code transplantation or integration with portable detection hardware. These missing steps prevent direct deployment for real-time industrial seed purity inspection.

Future work will focus on dataset expansion, multi-source feature fusion, lightweight model optimization and practical engineering deployment.

To begin with, we will build multi-condition hyperspectral datasets that cover diverse production regions, harvest years and seed storage periods to enrich data diversity. More than 100 test samples for each variety will be collected to alleviate metric instability induced by insufficient test samples per category. Domain adaptation and transfer learning will be adopted to offset spectral shifts caused by environmental variation. Meanwhile, open-set recognition modules will be integrated, enabling both known-variety classification and discrimination of unknown germplasm.

Next, we will fuse spectral curves with hyperspectral spatial texture features to fully capture subtle phenotypic differences among genetically similar rice seeds. Model pruning and quantization will be performed on the HHO-XGBoost architecture to cut computational burden. The full analytical workflow will be packaged into graphical software compatible with portable field-testing devices, facilitating industrial adoption.

Additionally, we plan to quantify seed internal biochemical properties including moisture, protein and starch content. This allows us to establish explicit correlations between SPA-selected sensitive bands and seed physicochemical traits, further uncovering the biological spectral response mechanism of japonica rice seeds. Moreover, the multi-stage optimization pipeline established in this work will be adapted for seed identification of wheat, corn and other grain crops, to validate its general applicability. Overall, this research provides solid technical support for crop germplasm conservation, seed quality supervision and the scaled promotion of elite rice varieties.

## 5 Conclusion

This study constructed a streamlined non-destructive identification framework based on hyperspectral imaging for distinguishing nine genetically similar japonica rice seed varieties. The workflow integrates multi-stage spectral preprocessing, optimal feature screening, interpretable analysis, and intelligent parameter optimization to achieve accurate germplasm classification. Mean spectral curves were adopted as model inputs, and Savitzky–Golay smoothing combined with Min–Max normalization was applied to suppress spectral noise and unify feature scales. The Successive Projections Algorithm effectively reduced high-dimensional spectral redundancy by selecting 22 low-collinearity sensitive bands while retaining varietal characteristic differences. SHAP analysis was further introduced to quantitatively interpret feature importance and sample-level classification contributions, addressing the inherent black-box limitation of conventional machine learning models. Optimized by the Harris Hawks Optimization algorithm, the XGBoost model achieved superior classification performance with an overall accuracy of 94.72% and a Kappa coefficient of 0.9389. Validated by per-class F1-scores, the proposed multi-strategy optimization framework significantly reduced misclassification among genetically similar rice varieties and improved identification stability. This study demonstrates that the combination of hyperspectral detection, SPA feature optimization, and HHO-based model tuning provides an effective non-destructive solution for the identification of closely related japonica rice germplasm, which can serve as a reliable technical reference for rice seed purity detection and crop germplasm resource identification.

## Data Availability

The original contributions presented in the study are included in the article/supplementary material, further inquiries can be directed to the corresponding author/s.
